# Annotation of cell types (ACT): a convenient web server for cell type annotation

**DOI:** 10.1186/s13073-023-01249-5

**Published:** 2023-11-03

**Authors:** Fei Quan, Xin Liang, Mingjiang Cheng, Huan Yang, Kun Liu, Shengyuan He, Shangqin Sun, Menglan Deng, Yanzhen He, Wei Liu, Shuai Wang, Shuxiang Zhao, Lantian Deng, Xiaobo Hou, Xinxin Zhang, Yun Xiao

**Affiliations:** College of Bioinformatics Science and Technology, Harbin Medical University, Harbin, 150086 China

**Keywords:** Single-cell RNA sequencing, Cell type annotation, Hierarchically organized marker map, Cell type enrichment method, Easy-to-use web server

## Abstract

**Background:**

The advancement of single-cell sequencing has progressed our ability to solve biological questions. Cell type annotation is of vital importance to this process, allowing for the analysis and interpretation of enormous single-cell datasets. At present, however, manual cell annotation which is the predominant approach remains limited by both speed and the requirement of expert knowledge.

**Methods:**

To address these challenges, we constructed a hierarchically organized marker map through manually curating over 26,000 cell marker entries from about 7000 publications. We then developed WISE, a weighted and integrated gene set enrichment method, to integrate the prevalence of canonical markers and ordered differentially expressed genes of specific cell types in the marker map. Benchmarking analysis suggested that our method outperformed state-of-the-art methods.

**Results:**

By integrating the marker map and WISE, we developed a user-friendly and convenient web server, ACT (http://xteam.xbio.top/ACT/ or http://biocc.hrbmu.edu.cn/ACT/), which only takes a simple list of upregulated genes as input and provides interactive hierarchy maps, together with well-designed charts and statistical information, to accelerate the assignment of cell identities and made the results comparable to expert manual annotation. Besides, a pan-tissue marker map was constructed to assist in cell assignments in less-studied tissues. Applying ACT to three case studies showed that all cell clusters were quickly and accurately annotated, and multi-level and more refined cell types were identified.

**Conclusions:**

We developed a knowledge-based resource and a corresponding method, together with an intuitive graphical web interface, for cell type annotation. We believe that ACT, emerging as a powerful tool for cell type annotation, would be widely used in single-cell research and considerably accelerate the process of cell type identification.

**Supplementary Information:**

The online version contains supplementary material available at 10.1186/s13073-023-01249-5.

## Background

Single-cell RNA sequencing (scRNA-seq) is widely used to analyze cellular heterogeneity by profiling thousands of individual cells in a single experiment. It provides unprecedented opportunities to compile single-cell atlases, identify novel and rare cell types and states, reveal intracellular and intercellular interactions, and characterize microenvironment composition, which is revolutionizing our understanding of cell biology and bringing new insights into the dynamic processes of complex ecosystems in healthy and dysfunctional tissues.

One basic and indispensable step for interpreting scRNA-seq data is cell type annotation, which in general includes two main approaches: manual and automatic cell annotation. For manual annotation, cells are first clustered with unsupervised methods, and cell type identities are then manually assigned to cell clusters by matching cluster-specific upregulated marker genes with prior knowledge of cell-type markers. Automatic cell annotation classifies cells based on markers’ characteristic expression patterns [1, 2] or transfers cell type labels from reference data to query data through machine learning methods [3–6]. Automatic methods are efficient approaches for assigning labels to cells or clusters with high speed and reproducibility, especially when reliable known markers and high-quality reference datasets are available [7]. These automated cell type identification methods in general perform well for scRNA-seq datasets [8], while annotation of very similar cell types [9] or certain cell subtypes with deep annotation levels [8, 10] remains a challenge.

Expert manual annotation is still considered the gold standard method for cell type assignment [7]. Manual curation of cell markers and investigation of gene expression patterns leave researchers with a vivid understanding of cell types and deeply portray the characteristics of different cell types. With the help of professional knowledge, researchers can correctly identify cell types and even uncover potential new cell types or cell states in cell clusters that are fuzzy and difficult to determine. However, manual annotation is labor-intensive, requires expert knowledge, and heavily depends on the prior biological knowledge of cell-type markers.

Here, we developed ACT, a one-stop computing and analysis platform that considerably improves the efficiency of cell type annotation and helps users quickly and comprehensively understand and determine cell types.

### ACT features three key parts

#### Marker maps with hierarchical structure

We manually curated cell marker entries that were widely scattered in about 7000 single-cell publications, summarized the prevalence of canonical markers, and organized tissues and cell types into sophisticated ontological structures in human and mouse.

#### Weighted and integrated gene set enrichment method

Based on the hierarchically organized marker map, we developed a computational method for enriching cell types, which requires only a list of upregulated genes for cell clusters.

#### Well-designed charts and detailed statistical information

We developed a user-friendly and convenient web server showing rich charts and statistics to assist users in identifying cell types quickly and accurately.

## Methods

### Collecting cell marker entries

After using keywords to search single-cell articles of human and mouse in PubMed, we manually collected and sorted out more than 22,000 cell marker entries from about 7000 publications. Detailed information, such as PMID, species, tissue types, cell types, disease status, list of canonical markers used for cell annotation, and differentially expressed genes (DEGs, ranked by log2 fold-change in decreasing order or other measures in the order of most significant to less significant) specific to cell types, were collected. We carefully curated the canonical markers that were explicitly used to annotate, identify, and distinguish cell types in scRNA-seq studies. Markers that did not have a clear correspondence with cell types in the articles and Supplementary materials were excluded. For DEGs, we kept the results of differential expression analysis between each cluster and the rest of the clusters. DEG lists lacking confident annotations in the original literatures, as well as those for which gene rankings could not be obtained, were omitted. Each cell marker entry was double-checked by another researcher with domain expertise, and the incorrect or problematic entries were removed after a secondary verification based on the reference articles.

**Fig. 1:**
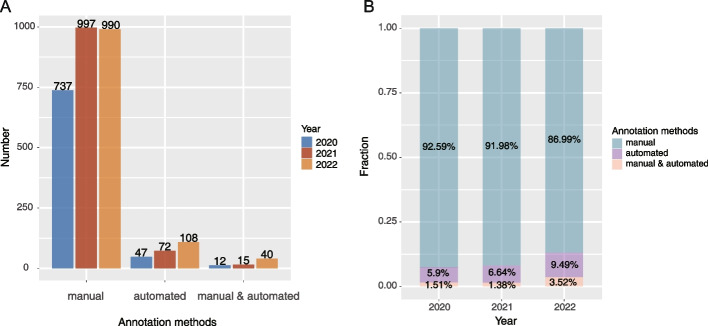
An overview of cell type annotation methods used in the scRNA-seq analysis in recent 3 years. **A** The number of publications with recorded cell type annotation approaches. **B** The relative percentage of frequency of cell type annotation methods in each year. Manual and automated: a combination of manual and automatic annotation

The mentioned methods concerning cell type annotation were manually extracted and then summarized (Fig. 1). Subsequently, the existing cell marker entries in databases, such as the CellMarker database [11], and multiple single-cell atlases [12–15] were integrated with collected entries.

### Unifying cell marker entries

We built a framework to standardize tissue names and cell-type names (Additional file 1: Fig. S1). For all tissue names, any misspelled entries were manually corrected and then mapped to the hierarchies of Uber-anatomy Ontology [16]. Furthermore, the tissue hierarchies were expanded to include some tissues that have not been covered by the Uber-anatomy Ontology. Regarding cell types, we used the following steps to standardize their diverse names: (1) correction of misspelled cell names; (2) conversion of abbreviated cell types to the full names; (3) removal of the broader cell types, such as “Immune cell” and “Hematopoietic cell”); (4) mapping the cell types to the Cell Ontology [17], by taking into account the tissue context. In addition, we added common cell types that were not present in the Cell Ontology to our cell-type hierarchies.

Marker genes of human and mouse were matched to the standard gene symbols, HGNC [18] and MGI [19], respectively. Typographical errors and inconsistent capitalization of genes were manually corrected by referring to the original studies. Genes that had no standard symbols or could not be regularized were filtered out.

### Generating tissue-specific cellular hierarchies and marker map

**Fig. 2:**
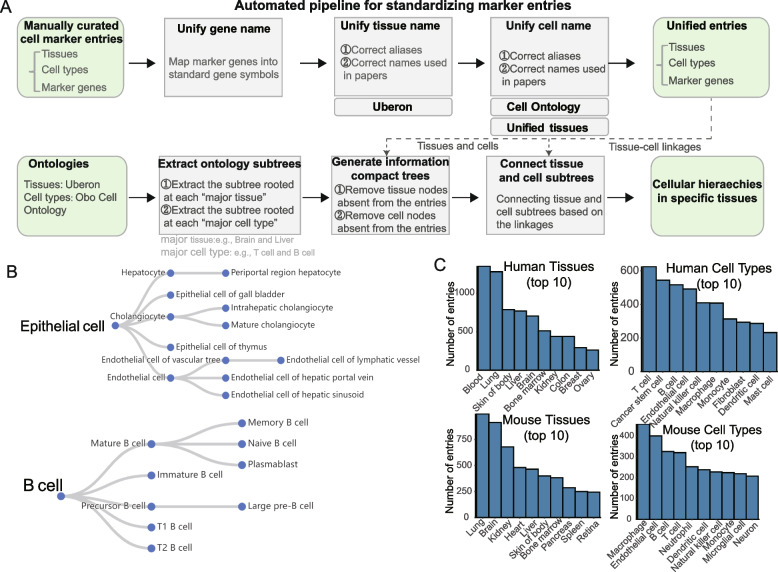
Construction of the marker map. **A** A standard framework to unify the tissue names, cell type names, and marker genes. **B** The epithelial cell and B cell lineages in human liver tissue. **C** The number of marker entries for tissues and cell types in human and mouse

Taking some important cell types (such as T cell and other “common-sense” cell types) as roots, the subtrees were thus extracted from the Cell Ontology. To make a compact presentation of the information in cell subtrees, we only retained the cell nodes that overlaid with recorded cell types in unified cell marker entries collected from the same tissue, and the following series of child nodes were connected to the nearest parent cell types. Similar to cell subtrees, we extracted the subtrees from the Uber-anatomy ontology with “common-sense” tissues (e.g., brain, liver) as the roots and only kept the tissues mentioned in unified cell marker entries. To generate tissue-specific cellular hierarchies, we connected each tissue with the cellular hierarchies which covered all cell types of the cell marker entries collected from the corresponding tissue (Fig. 2A).

Canonical markers for each cell type within each tissue were integrated by taking the union, and the frequency of each marker was summarized. For DEG lists of the same tissue and cell type, we employed the Robust Rank Aggregation method [20] to calculate a *p* value for each gene by aggregating the ranks across studies and applied multiple testing corrections on these *p* values. The genes were then ranked based on their adjusted *p* values, and a maximum of 3000 genes, for longer lists, were kept. Finally, an integrated gene list for each cell type under each tissue was retained.

To construct the pan-tissue marker map, the same procedure was employed with additional criteria: (1) cell types appeared in more than two tissues; (2) cell types with a total number of entries greater than 5; (3) more specific cell types were grouped into general cell types (e.g., entries of the epithelial cell of lung were integrated into epithelial cell entries).

### A Weighted and Integrated gene Set Enrichment method (WISE) for cell type annotation

We developed WISE to associate the input cell clusters with hierarchically organized cell types in the marker map. Firstly, for the input cluster differentially upregulated genes (DUGs), a weighted hypergeometric test (WHG) [21] was used to evaluate if the input genes (the list of genes of interest) are overrepresented in canonical markers associated with specific cell types (i.e., functional gene sets) in the marker map. Since markers with high usage frequencies typically signify greater reliability in cell type annotation, in this process, canonical markers were weighted based on their usage frequency, rendering that frequently used markers contribute more to the hypergeometric significance. In detail, for a specific cell type $c$, ${M}_{c}$ is its canonical marker set, and ${w}_{i}$ is the weight of gene ${g}_{i}$. If ${g}_{i}\in {M}_{c}$, ${w}_{i}$ equals to the usage frequency of gene ${g}_{i}$ in cell type $c$, and otherwise ${w}_{i}$ equals to 0. Further, we normalized the weight of all genes to have an average of 1 to keep the consistency of the weighted hypergeometric test and the conventional hypergeometric method [21]. For a cluster-specific input gene set (DUGs) $X$, the overrepresentation of $X$ in marker set (${M}_{c}$) is quantified as below:

1 $${P}_{whg}=\sum\limits_{a=k+1}^{\mathit{min}(m,n)}\frac{\left(\begin{array}{c}m\\ a\end{array}\right)\left(\begin{array}{c}N-m\\ n-a\end{array}\right)}{\left(\begin{array}{c}N\\ n\end{array}\right)}$$

$$N=\sum\nolimits_{g_i\in G}wi^Q,\;n=\sum\nolimits_{g_i\in X}wi^Q,\;m=\sum\nolimits_{g_i\in M_c}wi^Q,\;k=\sum\nolimits_{g_i\in K}wi^Q$$

where $G$ is the set of all protein-coding genes, $K$ is the set of overlap genes between $X$ and ${M}_{c}$. $Q$ is a power scaling factor. When $Q>1$, the difference among weights will be amplified. Based on prior experience, $Q$ is set to 3 by default [21]. To address the issue of non-integer calculations in the weighted hypergeometric computation, the gamma function was employed to generalize the calculation of the factorial function for non-integral values.

Furthermore, the GSEA method [22] was used to calculate the enrichment of the input gene set $X$ over the DEGs of cell type $c$, and the significance of the enrichment, ${P}_{gsea}$, was obtained. During GSEA, only the positive enrichment results (with positive NES values) were considered.

Finally, the weighted hypergeometric test (${P}_{whg}$) and GSEA analysis (${P}_{gsea}$) were combined by Fisher’s method. The combined *P* value was adjusted via the Benjamini–Hochberg method.

### Systematic evaluation of WISE

Five datasets were chosen as gold standard references. Two datasets containing FACS-sorted data, including the Tabula Sapiens [13] dataset with a broad range of human tissues and cell types (only manually annotated donor1 and donor2 were selected), and the human 10X PBMC 8k [23]. The rest three were annotated manually by experts, including the human liver [24], mouse lung [25], and mouse retina datasets [26]. All datasets have undergone detailed cell-type annotation in the original studies. All annotation results of these datasets have been manually inspected, and a few originally incorrect clusters were reannotated, and cell clusters mixed with multiple cell types were removed (Additional file 2: Fig. S2 and Additional file 3: Table S1). Finally, the gold standard datasets consist of 182 cell types across 17 tissues.

To make annotation results from different tools comparable, we created a catalog of major cell types through incorporating 51 common major cell types [27] and supplemented the catalog with additional specific cell types that were not initially included. Then the cell types in the catalog were aligned with our standardized cell types. The catalog encompassed a total of 176 major cell types (Additional file 3: Table S2), such as endothelial cell, B cell, and memory T cell. All predicted cell types were unified according to our standardized cell types and mapped onto the catalog for subsequent comparison.

To compute accuracy, we compared the most significant prediction (top 1) from each tool with the true label. If the predicted label is the same as the true label, or the predicted label is a subtype of the true label, based on the hierarchical structure of the cell tree, the prediction is deemed correct. The accuracy of prediction was calculated as the proportion of correctly predicted labels relative to all labels. The top 30 DUGs from each cluster are input into WISE, as it would be sufficient to obtain an accurate annotation result (Additional file 1: Fig. S3).

### Implementation

The frontend interfaces of ACT were implemented by Bootstrap, Struts2, JavaScript, and JSP. The common web technologies of highcharts.js, echarts.js, d3.js, datatables.js, zingchart.js, and ztree.js provided power for the interactive applications including summary tables, graphs of interactive hierarchy map, charts of markers and cell types, and cell lineage browser. The backend computational module of ACT was developed by the R program. The cell marker entries were stored in a MySQL database.

### Integrating large-scale scRNA-seq data

On the ACT, single-cell expression data and cell type annotation information from the HCA (https://data.humancellatlas.org), Tabula Sapiens [13], MCA [14], HCL [15], and the study of Emont et al. [28] were manually curated to provide vivid visualization of the expression of cell-type marker genes.

### Data cohorts of three case studies

In the first case study, we utilized a dataset comprising ~3k frozen peripheral blood mononuclear cells (PBMCs) from a healthy donor. These cells were classified into 9 clusters, including T cells, B cells, and monocytes. scRNA-seq profile was obtained from the 10X Genomics, and corresponding cell labels, which had been previously described by Zheng et al. [23], were also obtained. For case study 2, we focused on a cohort of 24 samples derived from 11 individuals diagnosed with basal cell carcinoma which were collected both before and after PD-1 blockade therapy. The dataset was sourced from the GEO data repository under accession of GSE123814 and contained 53,030 malignant, immune, and stromal cells [29]. Case study 3 involved an extensive cohort consisting of 122 samples from 42 patients, which integrated five independent scRNA-seq datasets. The samples were collected from various anatomic sites within the bilateral colon. This rich dataset, including more than 235,000 cells, was retrieved from the Synapse database under accession code syn26844071 [30].

### Single-cell data analysis in case studies

For all three case studies, expression data together with cell labels (if available) were downloaded from public repositories and then processed using Seurat (v4.2.0) with default parameters. Cluster-specific upregulated genes were calculated by the FindAllMarkers function, and up to the top 30 DUGs in each cluster were selected as the input. Plots and in-house R scripts could be available under reasonable request.

## Results

### An overview of cell type annotation methods

We first evaluated scRNA-seq studies in recent 3 years to obtain an overview of the usage frequency of methods about cell type annotation. We carefully went through over 5200 publications and corresponding Supplementary materials from PubMed (from the beginning of 2020 to September 25, 2022) to manually extract recorded cell type annotation methods in the scRNA-seq analysis of human and mouse. We found that about 90% of publications typically assigned cell identities by manual annotation (Fig. 1A, B), and a little part of studies took alternative ways: automatic cell annotation or a combination of manual and automatic annotation. In the past 3 years, manual annotation is still the most popular approach (Fig. 1B), even though the automatic cell annotation method is increasingly used.

### Construction of a marker map in human and mouse

These large numbers of single-cell studies that annotate cell types by manual-based approach have successfully identified a wide range of cell types in various tissues based on abundant cell-type markers. During the manual annotation procedure, markers with a strong ability to identify cell types are frequently used, while the weaker ones are relatively seldom used. For instance, in human blood tissue, important B cell markers [31] such as *MS4A1* (encoding CD20), *CD79A*, *CD79B* (components of the B cell receptor), and *CD19* (a surface marker) ranked among the top four in usage frequency. Similarly, highly specific T cell markers *CD3D*, *CD3E*, and *CD3G* [32], and NK cell markers *NKG7*, *GNLY*, *NCAM1*, and *FCGR3A* [33] all exhibited the highest usage frequency (Fig. S4). Consequently, the prevalence of marker genes has become an important tacit knowledge about choosing suitable and correct markers of specific cell types, which has become a potential consensus among researchers. Moreover, a rich and complex hierarchy comprising cell types and their subtypes provides a structural foundation for achieving a refined annotation of cell types. Therefore, integrating cell-type marker genes from existing literature, establishing the usage frequency spectrum of these markers, and constructing the cellular hierarchies will provide important support for rapid and precise cell type annotation.

We manually curated 26,785 cell marker entries from about 7000 publications, existing databases [11], and multiple single-cell atlases [12–15]. To unify the confusing and cluttered cell marker entries from extensive and various sources, we constructed a standard framework (see “Methods”) to unify the tissue names, cell type names, and marker genes (Fig. 2A). By organizing the cell types of cell marker entries into a series of cellular hierarchies, we then generated tissue-specific cellular hierarchies. For example, in human liver tissue, a total of 27 cellular hierarchies, such as epithelial cell lineage, B cell lineage, were included (Fig. 2B). To obtain the prevalence of cell-type-specific markers, the marker genes were aggregated to count their usage frequencies in each cell type of each hierarchy. In addition, the ranked DEGs for each cluster, which might serve as a potential novel marker to further refine cell type annotation, were collected and integrated. Finally, a comprehensive and standard cell marker map was constructed by integrating the cellular hierarchies, the cell-type-specific marker genes with usage frequency, and the differential gene lists. The marker map was composed of 23,826 unified cell marker entries, involved a total of 4197 marker genes, 4419 differential gene lists, and 2040 cellular hierarchies derived from 806 cell types of 282 tissues in human, and included a total of 7955 marker genes, 3271 differential gene lists, and 1627 cellular hierarchies derived from 867 cell types of 240 tissues in mouse (Fig. 2C). Compared with released cell marker resources, CellMarker [11], PanglaoDB [34], clustermole [35], and MSigDB [36], our marker map contains a broader range of tissue and cell types (Additional file 1: Supplementary methods).

### A weighted and integrated gene set enrichment method for cell type annotation

**Fig. 3:**
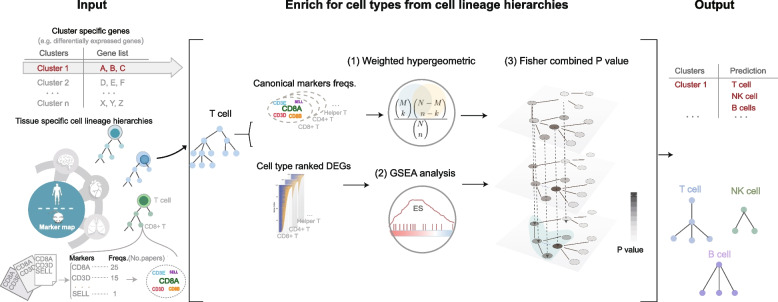
Overview of the WISE method for cell-type assignment

To annotate cell types for cell clusters, we directly used differentially upregulated genes of clusters to determine whether they are significantly overrepresented in the cell-type-specific canonical and potential markers. Since the well-established marker map contained both cell-type canonical markers that are typically used for precise cell-type identification and cell-type-related differentially expressed genes that can serve as potential markers, we sought to combine the two types of markers to improve the accuracy and reliability of cell-type assignment. Inspired by the thought of functional enrichment analysis, we developed a Weighted and Integrated gene Set Enrichment method (WISE) to assign cell types to cell clusters (Fig. 3).

### Systematic benchmarking of WISE

To benchmark the performance of WISE, we compared it against seven automated cell type annotation tools, including scType [37], scSorter [38], SCINA [2], SCSA [39], CellAssign [1], EasyCellType [40], and scCATCH [41]. The evaluation of the accuracy of WISE was performed using five distinct datasets, including the Tabula Sapiens (a multi-tissue dataset) [13], human liver [24], human PBMC [23], mouse lung [25], and mouse retina [26]. Noteworthy, during the evaluation process, markers from all five datasets were not included in the marker map. Regarding the evaluation results, the most significant (top one) result was deemed as the predicted label. To ensure the comparability of predicted cell types across the tools, a major-cell-type catalog was created (covering 176 major cell types, such as memory T cell, B cell, and endothelial cell), and all predicted cell labels were mapped to the corresponding cell types in the catalog (see “Methods”).

**Fig. 4:**
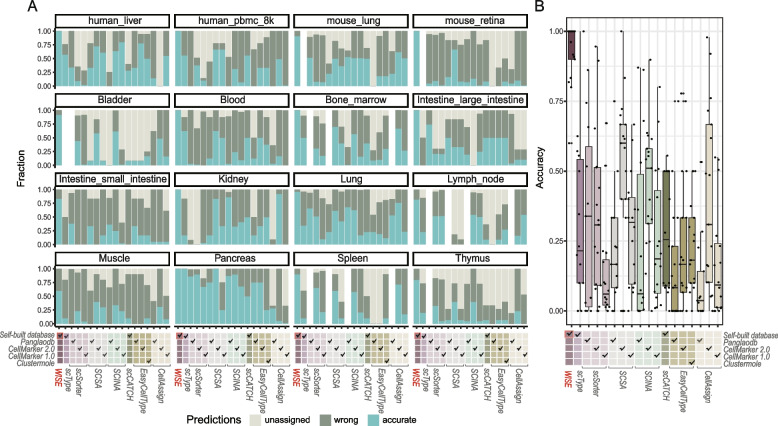
Benchmarking the performance of WISE against seven methods in five datasets. **A** The fraction of accurately predicted clusters, incorrectly predicted clusters, and unassigned clusters in the predictions made by all tools across five datasets. Different cell marker databases were used as input for each tool, including self-built databases and publicly available databases like PanglaoDB, CellMarker 1.0, CellMarker 2.0, and clustermole. The last 12 tissues were from the Tabula Sapiens dataset. The results for Lung_Trachea were not shown as this tissue was absent in many cell marker databases. **B** Accuracy comparison for all methods

We then comprehensively assessed the performance of each method in terms of the numbers of correctly predicted clusters, wrongly predicted clusters, and unassigned clusters within each tissue of each dataset. We found that WISE had the highest proportion of correctly predicted cell types, and the lowest proportion of prediction error and unassigned labels, in all datasets and all tissue types (Fig. 4A and Additional file 1: Fig. S6A). Furthermore, accuracy was calculated based on the proportion of correctly predicted labels, and WISE achieved the highest accuracy in all datasets, reaching an average accuracy of 92.6% (Fig. 4B). Taking the human liver dataset as an example, WISE achieved 100% accuracy in this dataset, and each predicted label was concordant with its canonical markers’ expression (Fig. S5). In contrast, other methods exhibited relatively low average accuracy, ranging from 14.2 to 80.1%, along with a high proportion of unassigned labels (ranging from 0 to 74%), as well as a high proportion of incorrectly predicted labels (ranging from 12 to 85.8%).

Next, we evaluated the contribution of weights (the usage frequency of canonical markers) to WISE by comparing the performance of WISE with weights, without weights, and with permutated weights. We found that introducing the usage frequency of markers as weights significantly enhanced the performance of WISE (Fig. S6B, $P=2.8\times {10}^{-15}$, paired Wilcoxon rank sum test).

### Annotation of cell types (ACT), a one-stop platform for annotating cell types

**Fig. 5:**
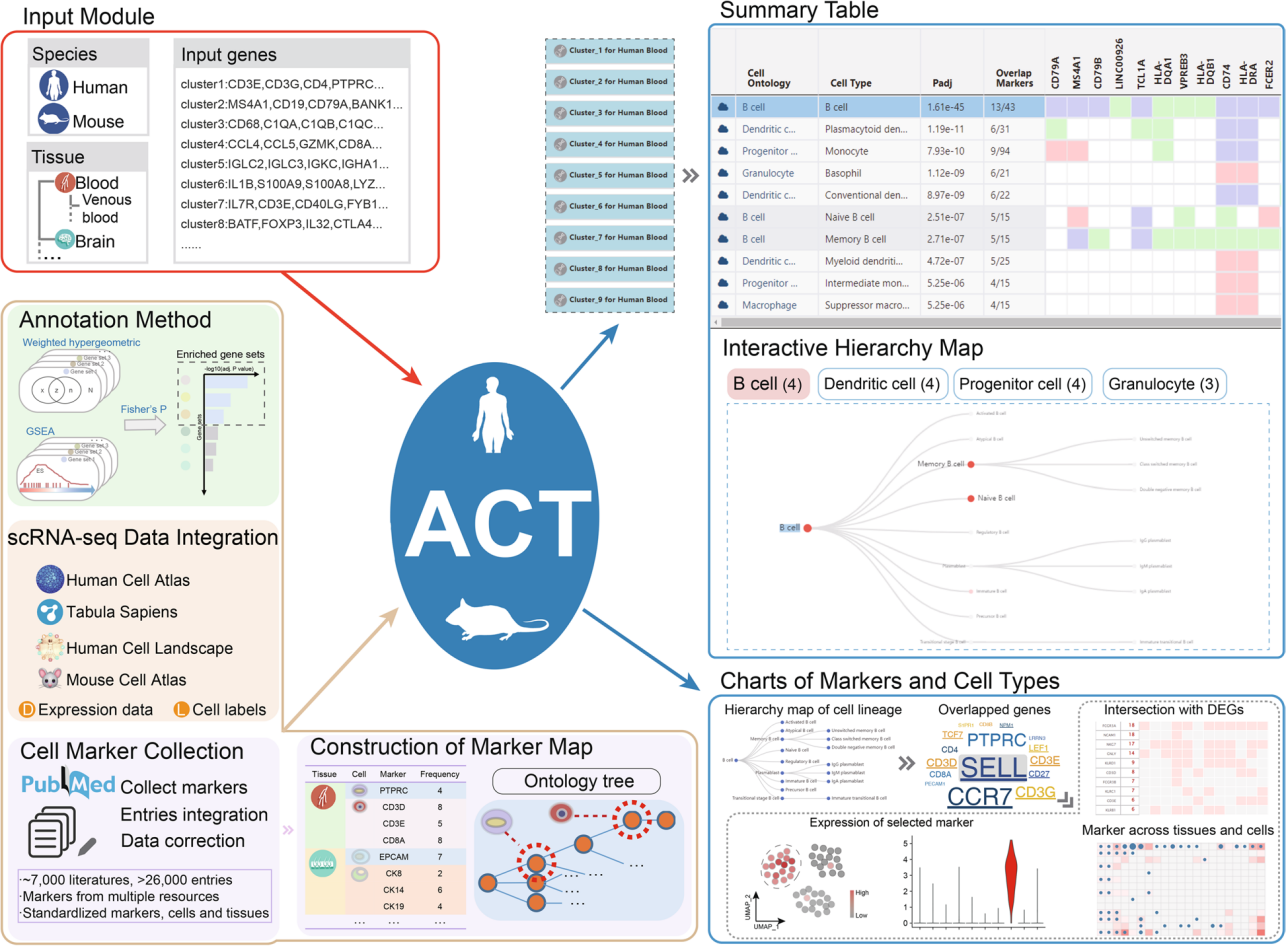
Schematic workflow of ACT web server

Based on the marker map and the proposed cell type annotation method (see “Methods”), we developed a one-stop cell annotation platform ACT (http://xteam.xbio.top/ACT/ or http://biocc.hrbmu.edu.cn/ACT/) with the aim of efficiently annotating cell types (Fig. 5). Users are able to explore and annotate cell clusters of interest via convenient and easy-to-use modules implemented in ACT, including four main components: (1) the input module (red box) to accept a list of upregulated genes from cell clusters, (2) the output summary table (top right blue box), (3) the interactive hierarchy map (top right blue box) of enriched cell types, (4) the marker prevalence of specific cell types under cell lineages (bottom right blue box) with user-friendly interfaces and ready-to-use functionalities showing panels of rich charts and statistics. The core data of ACT (golden box) is composed of the structural marker map and integrative large-scale single-cell transcriptome atlas. The WISE method (golden box) is used to enrich candidate cell types organized by the cellular hierarchies in the ACT.

### Input module

To perform an ACT task, ACT simply takes a list of ordered DUGs for cell clusters as input. Users should also select species (“Human” or “Mouse”) from the pulldown menu and tissue types from the hierarchical tree of tissues on the home page (Fig. 5, “Input Module”).

### Summary table

Upon clicking the “Submit” button, cell type annotation is performed. The result page beneath the input module is displayed as a list of collapsible title bars within a few seconds. Each title bar matches the annotation results for each input gene list, and the first one is unfolded by default. To obtain an overview of enriched cell types, entries and statistics are mainly presented in a summary table (Fig. 5, “Summary Table”), including information on cell types (column “Cell Type”) and corresponding lineages (column “Cell Ontology”), Benjamini–Hochberg-corrected *P* values (column “Padj,” *q* values), numbers of overlapped genes between the input DUGs and cell-type markers (column “Overlap Markers”), and an embedded heatmap (genes in the columns are of the same order as user-input DUGs) showing the intersection of input DUGs with canonical marker genes (red), DEGs (green), or both (purple). Each row of the summary table/heatmap is for one enriched cell type. The tabulated list will be arranged in ascending order according to the significance (column “Padj”), where only the most significantly enriched cell type (top 1) is displayed by default. Based on our evaluation results (Fig. 4), we recommend selecting the “top 1” option, as it can effectively annotate cell types. The top ten annotation results for each cluster can also be alternately obtained.

### Interactive hierarchy map

To further incorporate the structural hierarchy of cell types for elaborately annotating cell types, hierarchy maps of enriched cell types are constructed and shown as cell lineage trees in the selected tissue (Fig. 5, “Interactive Hierarchy Map”). It should be noted that if the users select to display the 10 significantly enriched cell types, the interactive hierarchy map can be useful. These tree views in it provide users with a global perspective to compare the enriched cell types at different resolution levels. The color, size, and label size of nodes in the cell lineage trees are set according to the rank of the adjusted *P* values in ascending order. When users click cell lineages of enriched cell types in the summary table, the tree will automatically switch among cell lineages, and the selected item is also highlighted.

### Charts of markers and cell types

To provide detailed and comprehensive information for understanding the characteristics of cell types and markers, ACT offers a series of charts for cell types and markers which can be triggered by clicking the “cloud” icon at the beginning of each row and colored cells of the heatmap in the summary table (Fig. 5, “Charts of Markers and Cell Types”). The prevalence of canonical markers is summarized into word clouds (with DUGs underlined). A large amount of expression data integrated from several single-cell atlases and visualization of additional empirical data generated by manually curated entries are used to help confirm whether marker genes can effectively mark cell identities.

### BatchACT

Furthermore, ACT allows users to submit multiple clustering results and helps them determine the optimal clustering result. ACT will annotate all cell clusters for each clustering result. Subsequently, a plot is generated to depict the variation in the number of unique cell types and in the ratio of unique cell types to all cell clusters across different clustering results. The ratio of unique cell types to all cell clusters represents the non-redundancy of annotated results, with higher values indicating more clusters are assigned to unique cell types. This plot will help users choose optimal clustering parameters which reach a balance of a high number of unique cell types and a high ratio of non-redundant cell types (Additional file 1: Fig. S7).

In addition, users can conveniently search and browse the cell type-specific markers on the “Search” page. All of the pictures, graphs, and tables produced by ACT are available for direct download and the “Download” page provides cell marker lists of each tissue. A detailed tutorial for the usage is also provided on the “Help” page.

### How to use ACT for cell type annotation: a case study based on human PBMC dataset

To demonstrate how to use ACT for cell type annotation, we re-analyzed a human peripheral blood mononuclear cell dataset, PBMC 3k [23]. The processed gene expression data, together with well-defined cell labels, including 9 cell types (Naive CD4+ T cell, Memory CD4 T cell, CD14+ Monocyte, B cell, CD8+ T cell, FCGR3A+ Monocyte, NK cell, Dendritic cell, and Platelet), was obtained from the 10X Genomics website, which profiled 2638 immune cells of frozen PBMCs from a healthy donor.

**Fig. 6:**
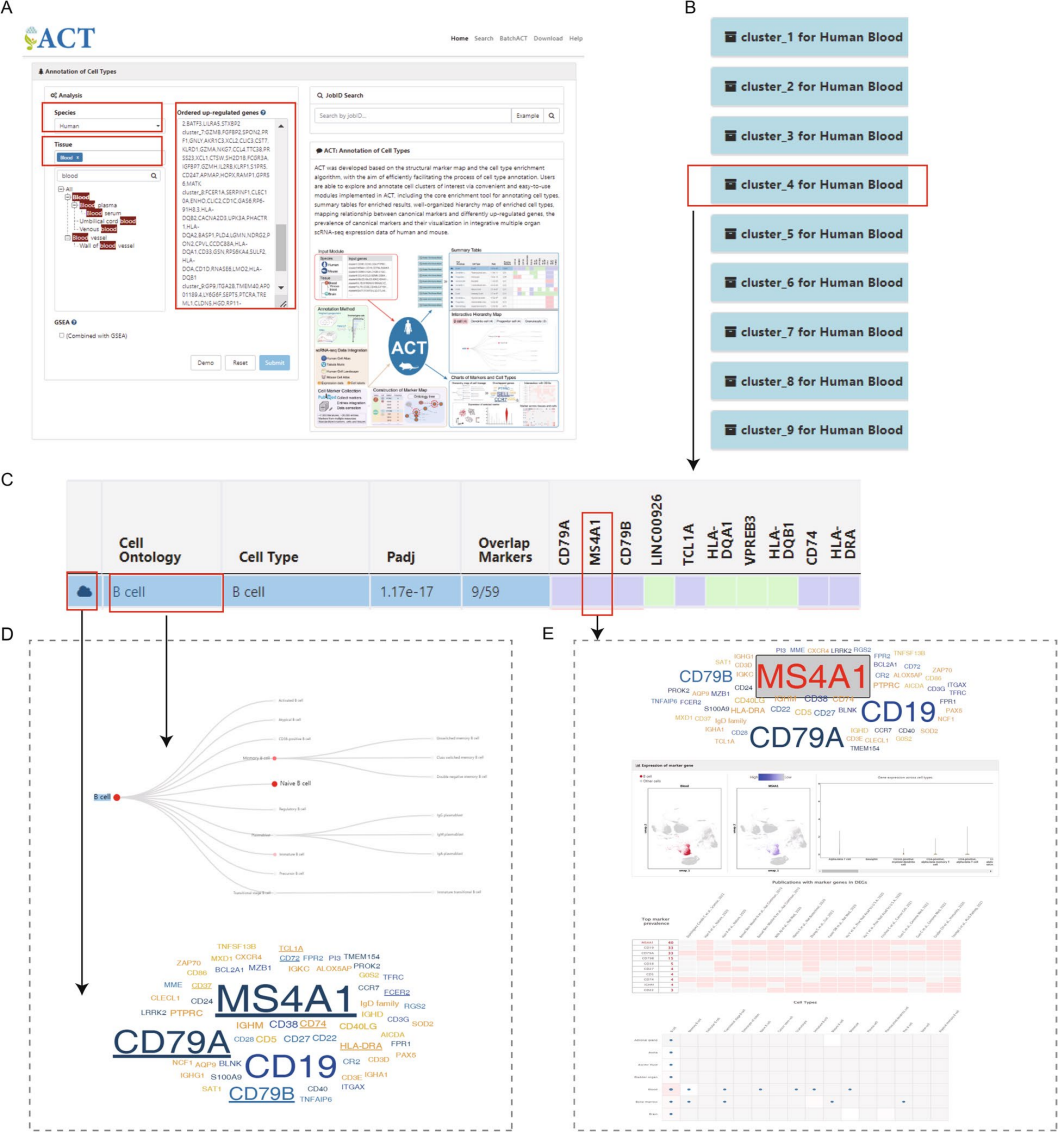
ACT provides well-designed charts and statistical information to assist in quickly and conveniently annotating cell types. **A** The example input for the human PBMC 3k dataset. **B** Overall annotation results for all 9 clusters. Each bar corresponds to the annotations for one cluster, and all but one of the title bars were folded by default. **C** Main summary table of B cell. **D** Word cloud and interactive hierarchy map triggered by clicking the “cloud” icon and “Cell Ontology,” respectively. **E** More details about marker genes of B cells

In the input module, species and tissue type were set as “Human” and “Blood,” respectively, and the gene list comprising the top 30 upregulated genes in each of the 9 cell types/clusters was used as input (Fig. 6A). The annotation results for all 9 clusters could be presented below the input panel in less than half a minute (Fig. 6C). On the result page of each collapsible title bar, the main summary table, comprising the well-tabulated statistics and an embedded heatmap (Fig. 6C, left panel), was positioned on the top. The most significantly enriched cell type was assigned to each cell cluster. Taking cluster 4 as an example, we observed that the B cell was the most significantly enriched cell type (Fig. 6C, adjusted *P* value ${P}_{adj}=1.17\times {10}^{-17}$). Furthermore, in the interactive hierarchy map of enriched entries under the summary table, B cell was the most distinguishable term in the tree of B cell lineage according to the size, color, and label size of nodes (Fig. 6D, top panel). After clicking the “cloud” icon at the beginning of an enriched cell type, a pop-up window with a word cloud was presented (Fig. 6D, bottom panel). In the word cloud, the size of genes indicated the usage frequency of markers, and marker genes overlapping with the input genes were underlined. We observed that the three top-ranked input genes (*CD79A*, *MS4A1*, and *CD79B*), have been widely used to identify B cells in blood tissue (Fig. 6D, bottom panel). More detailed information about these marker genes could be given on a new page by clicking the colored cells in the heatmap. Through interactively switching UMAP and violin plots of expression of marker genes, we confirmed that all of these three marker genes were specifically expressed in B cells of the blood tissue based on the integrated single-cell atlas of human (Fig. 6E, the second panel). Besides, two panels used to characterize the selected markers in specific cell types were shown (Fig. 6E, the third and fourth panels). The first panel showed how often and to what extent the canonical marker genes were identified as differentially expressed genes. We observed that *MS4A1* was frequently identified as a top-ranked upregulated gene by several studies (Fig. 6E, the third panel). And the second panel showed whether the selected canonical marker could also be used as a marker gene in a broad range of human tissues and cell types. We observed that *MS4A1* was also used as a cell-type marker (blue dots) in multiple tissues and cell types (Fig. 6E, the fourth panel). Using ACT, we quickly determined the identities of all cell clusters (Additional file 1: Fig. S8), and our annotation results were highly consistent with predefined cell labels (Additional file 1: Table S3).

### ACT achieved accurate and more sophisticated annotations: a case study in basal cell carcinoma

To explore the application and features of ACT, we performed cell type identification with ACT based on the transcriptomic profiling and paired cell type annotation results of a basal cell carcinoma (BCC) dataset [29]. In the original study, Yost et al. discovered different kinds of immune and stromal cells and defined two clusters of malignant cells in BCC which was common skin cancer and originated from keratinocytes near the basal layer of the epidermis.

**Fig. 7:**
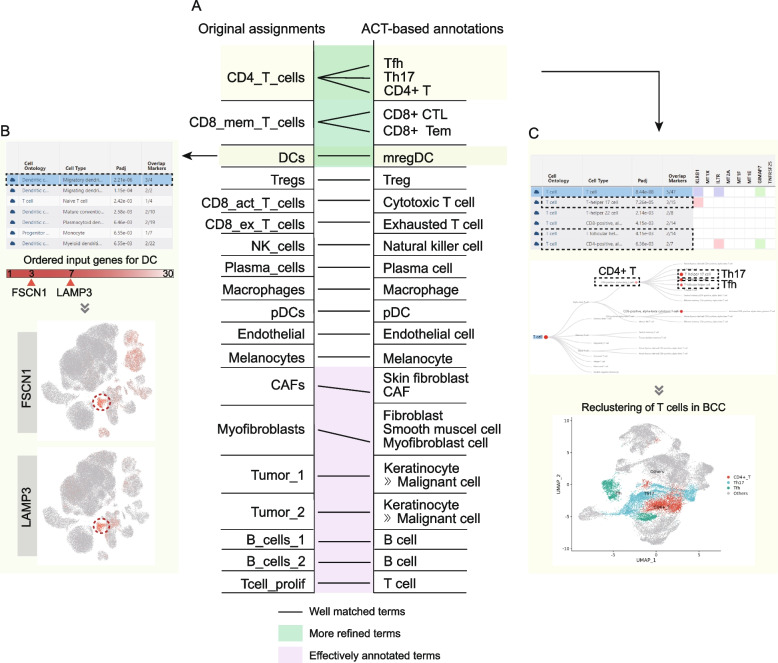
Manual cell annotations with ACT are accurate and more sophisticated in basal cell carcinoma. **A** Comparison between original cell type labels and annotation results based on ACT. **B** The summary table of ACT indicated that the original dendritic cell was actually migratory dendritic cell. High expression of top-ranked upregulated genes, *FSCN1* and *LAMP3*, in BCC is shown as evidence. **C** As shown in the summary table and interactive tree, the CD4+ T cell was a mixture of several similar cell types. Reclustering and annotation further confirmed this classification. Th17: T-helper 17 cell; Tfh: T follicular helper cell; CTL: Cytotoxic T cell; Tem: Effector memory CD8-positive, alpha-beta T cell; DC: Dendritic cell; mregDC: Migratory dendritic cell; Treg: Regulatory T cell; CD8_act_T_cells: CD8+ activated T cell; CD8_ex_T_cells: CD8+ exhausted T cell; pDC: Plasmacytoid dendritic cell; CAF: Cancer-associated fibroblast; Tcell_prolif: proliferating T cell

To perform an ACT task, we identified upregulated genes in each cell cluster and took the first 30 genes as input. Cell identities were then manually assigned by means of the annotation results from ACT. Compared with the previous assignments [29], our annotations were accurate and more sophisticated. All of the cell clusters (19/19) were correctly annotated, and high-frequency and canonical marker genes in the structural marker map were visualized to verify the annotation results (Fig. 7A, Additional file 1: Table S4 and Fig. S9). In the dendritic cell cluster, we observed the high expression of migration-associated marker genes *FSCN1* and *LAMP3*, thus it would be more appropriate to mark this cluster as migratory dendritic cell [42] (Fig. 7B). Besides, based on the mapping relationship between the cluster-specific input genes and cell-type-specific markers under the hierarchically organized marker map, the multiple-level and more refined cell types were uncovered (Fig. 7A, Additional file 1: Fig. S9 and Table S4). We found that the previously annotated CD4+ T cell cluster was indeed a mixture of CD4-positive, alpha-beta T cell, T-helper 17 cell, and T follicular helper cell [29] (Fig. 7C, top and middle panels). After reclustering all T cells and annotating cell types based on ACT, we further confirmed these cell types (Fig. 7C, bottom panel). Similarly, the original CD8+ memory T cell cluster could also be classified into effecter memory and cytotoxic cell subpopulations (Additional file 1: Fig. S9). In addition, cell identities of clusters were correctly identified even when the key canonical marker genes (e.g., *PDPN* and *FAP* of cancer-associated fibroblast) were out of the input gene list (Fig. 7A). Overall, with the aid of ACT, we could accurately and efficiently perform the annotation of cell clusters.

### A refinement of cell annotations in colon cancer: a case study of large-scale integrative analysis based on ACT

In this case study, we examined the capacity of ACT to address the annotation of single-cell data with tens of thousands of cells. Re-analysis was conducted on 235,000 cells in colon cancer, which integrated five independent scRNA-seq datasets [30].

**Fig. 8:**
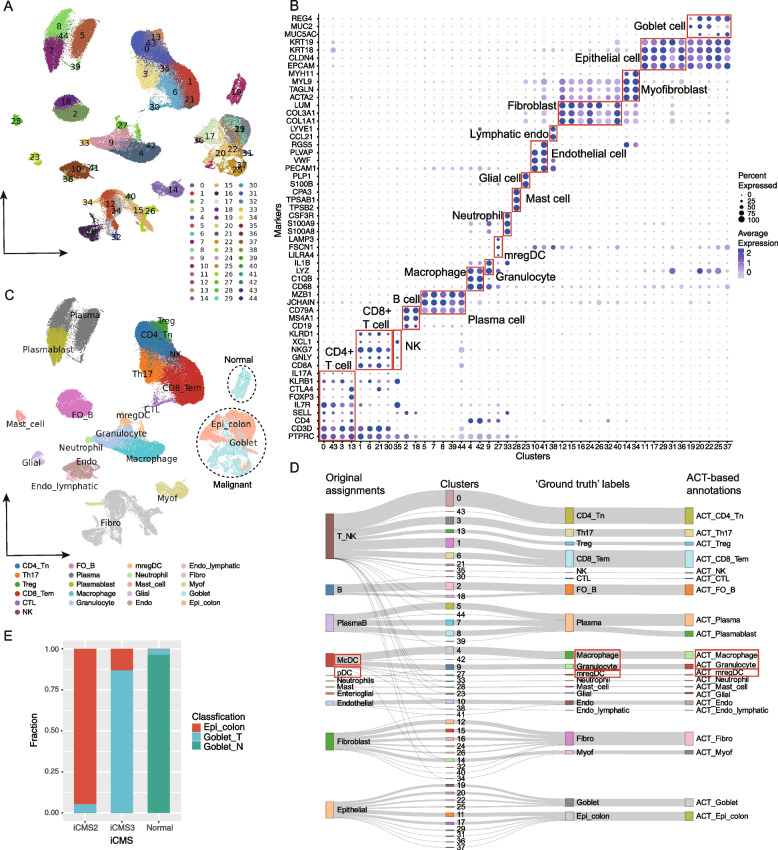
Refinement and annotation of cell clusters in colon tissues. **A** Reclustering of cells and its UMAP projection in colon tissues. **B** “Ground truth” cell cluster labels derived from manual cell annotation based on the marker map. **C** Annotation results based on ACT. **D** A Sankey diagram to show the comparison of annotation results. Cell types that might be mislabeled are shown in the red box. **E** Epithelial cells and goblet cells correspond to the iCMS2 and iCMS3 subgroups in the original publication, respectively. CD4_Tn, Naive thymus-derived CD4-positive, alpha-beta T cell; Th17, T helper 17 cell; Treg, Regulatory T cell; CD8_Tem, Effector memory CD8-positive alpha, beta T cell; CTL, Cytotoxic T cell; NK, Natural killer cell; FO_B, Follicular B cell; Plasma, Plasma cell; mregDC, Migratory dendritic cell; Glial, Glial cell, i.e., Entericglial cell; Endo, Endothelial cell; Endo_lymphatic, Lymphatic endothelial cell; Fibro, Fibroblast; Myof, Myofibroblast cell; Goblet, Goblet cell; Epi_colon, Colon epithelial cell; Goblet_T, Malignant goblet cell; Goblet_N, Normal goblet cell; McDC, Monocyte conventional dendritic cell; pDC, Plasmacytoid dendritic cell

The clustering analysis generated 45 clusters (Fig. 8A). Using ACT, we performed the annotation of cell types of these clusters. Compared with the manual annotation results, we found that ACT could quickly annotate cell clusters with high accuracy, and 100% (45/45) clusters were correctly identified (Fig. 8B–D and Additional file 1: Table S5). More subtle cell types were also discovered. For example, part of endothelial cells was identified as lymphatic endothelial cells (Fig. 8B, C, marked by *LYVE1*). Besides, a few cell types in the original annotations were corrected (Fig. 8D). The previously annotated plasmacytoid dendritic cells were identified as migratory dendritic cells based on the high expression of migration-related genes *FSCN1* and *LAMP3* (Fig. 8B–D). The monocyte conventional dendritic cell cluster was re-classified into macrophage and granulocyte.

In the originally annotated epithelial cell cluster, goblet cells were determined (because of the high expression of marker genes *MUC2* and *REG4*) (Fig. 8C). In line with our identification of a malignant goblet cell subtype in colon cancer cells, recent research by Uhlitz and colleagues [43] identified immature goblet cells among malignant cells in colorectal cancer patients. Besides, Hu et al. [44] discovered some malignant cell clusters exhibited high expression of goblet cell canonical markers, coupled with developmental trajectories closely resembling those of normal goblet cells, in mucinous adenocarcinomas of colorectal cancer. In malignant cells, Joanito et al. discovered and validated two functional subtypes (iCMS2 and iCMS3) in multiple scRNA-seq datasets and bulk transcriptomes. Notably, we found that the iCMS2 and iCMS3 subtypes were significantly enriched for malignant epithelial cells and goblet cells, respectively (Fig. 8E, ${\chi }^{2}$ test, $P<0.001$). We further observed that only the malignant goblet cells could be further divided into the microsatellite unstable (iCMS3_MSI) and microsatellite-stable (iCMS3_MSS) groups and the newly uncovered malignant epithelial cells barely contained MSS cells (iCMS2_MSS). These results suggest completely different malignant cell types underlying these two subtypes, highlighting the value of refined cell annotation based on ACT.

### Construction of the pan-tissue marker map allows for mapping cell types in less-studied tissues

For some tissues, the lack of cell-type markers and focused single-cell studies largely hinder the process of cell type annotation. While tissues or organs, with well-established cell type markers, may share common cell types, harbor conserved cell-type features and thus provide references for annotation in less well-studied tissues. Thereby, we build a pan-tissue marker atlas by fusing all cell marker entries from 282 human tissues and 240 mouse tissues separately, which includes 175 and 131 cell types in human and mouse, respectively.

**Fig. 9:**
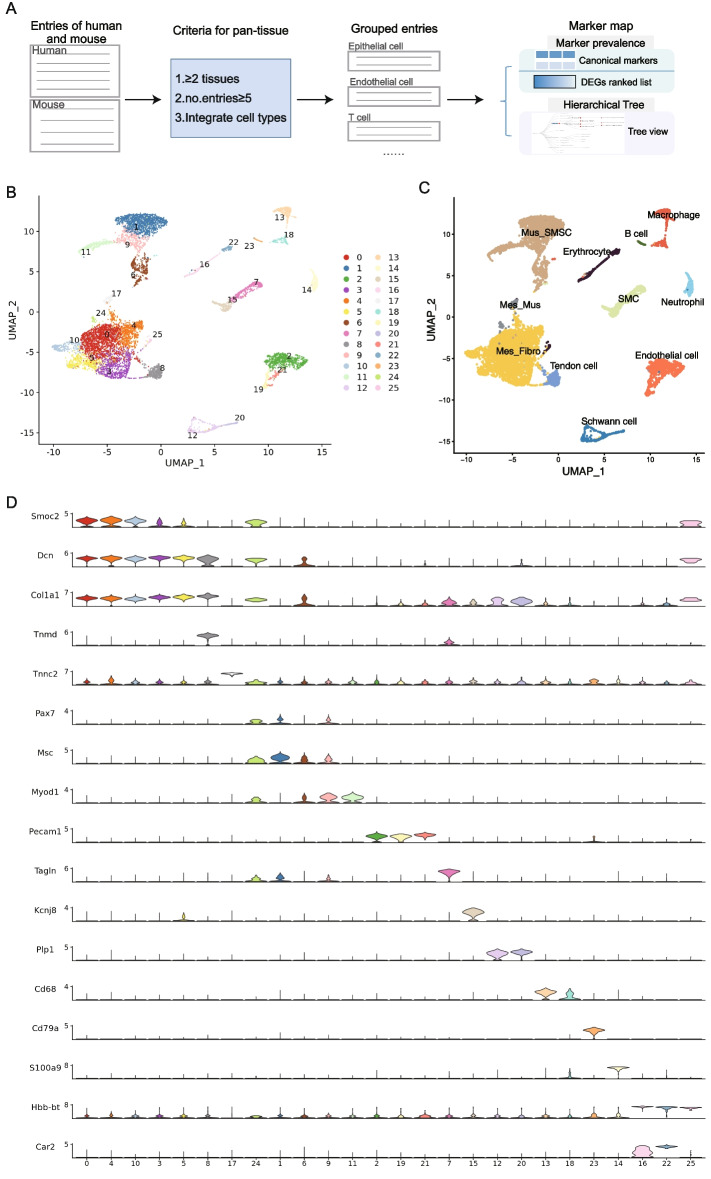
The pan-tissue marker map and its application. **A** Diagrammatic sketch to illustrate the construction of the pan-tissue marker map. **B** 26 clusters were obtained after clustering. **C** UMAP plot colored by assigned cell types based on ACT annotation results. **D** A stacked violin plot shows the expression of canonical markers in each cell cluster. Mes, mesenchymal cell; Fibro, fibroblast; Muc, muscle

To test the application of the pan-tissue marker map, we performed a re-analysis for a single-cell dataset of muscle-tendon junctions in mouse [45]. Using markers from the skeletal muscle tissue in ACT, we could annotate 20 (76.92%) of the total 26 cell clusters, during which two Schwann cell clusters were coarsely identified as glial cells. While based on the pan-tissue marker map, all cell clusters were successfully annotated (with accuracy >96%, Fig. 9B, C). Two immune cell clusters were divided into B cells and macrophages separately based on the high expression of *Cd79a* and *Cd68*, and the two Schwann cell clusters were also precisely annotated (Fig. 9B–D). Specially, the original “dual identity” cluster, which expressed myogenic (*Myod1*) and fibrogenic (*Col1a1*) markers, was identified as mesenchymal cell, fibroblast, or muscle cell (Fig. 9B–D). In short, our practical marker map of pan-tissue enables users to annotate cell types of scRNA-seq data in less well-studied tissues quickly and efficiently. We reason that further combining pan-tissue information with that of individual tissues could considerably enhance the accuracy of cell type annotation of specific tissues.

## Discussion

Manual cell type annotation, to our best knowledge, is still treated as the gold standard method [7, 12, 13, 15], but it’s labor-intensive and subjective. Automatic approaches require trained models and/or well-established reference datasets and can be technically demanding. And the performance heavily relies on the data quality and procedure of data processing, which can vary greatly among studies. Here, we presented ACT, a web server for quickly and efficiently annotating cell types and providing more convenience for assigning cell identities.

As an efficient and powerful tool for cell type annotation, ACT possesses four key features: the simple input, the comprehensive and hierarchically organized marker map, the weighted and integrated cell type annotation method, and the rich and convenient graphical interfaces. In contrast to several automatic cell annotation methods and tools that take the whole gene expression profile and/or reference scRNA-seq dataset to perform cell type annotation [3–5, 46], ACT only requires simple lists of genes (e.g., top upregulated genes in each cluster) as input. It is very convenient for users to perform cell type annotation as the input genes can be easily obtained from differential expression analysis. The marker map that derived from expert manual curation contains a wide spectrum of hierarchically organized cell types and provides users with better annotation practices while requiring less expert knowledge. Using the marker map, cell clusters can be annotated at different resolution levels, and the prevalence of marker genes in it supplies the prior knowledge of literature-supported cell type annotations. By integrating the canonical markers where their usage frequency is treated as the weights and the ordered cell-type-specific DEGs that serve as potential markers, we proposed the WISE method to rapidly and precisely associate each input cell cluster with cell types in the marker map, during which this integration further boosts the accuracy of cell type identification. To further facilitate the annotation and alleviate the dependence on prior knowledge of cell types and markers, ACT offers rich and well-designed graphical interfaces and statistics, such as the summary table, interactive hierarchy map, word cloud showing the summarized marker prevalence, plots of gene expression, etc. In cases where cell clusters cannot be annotated by ACT, these clusters could be identified as potential novel cell types after the researchers have precluded the possibility of noise clusters or mixed clusters.

In summary, ACT, an emerging powerful tool for annotating cell types, will be widely used and more efficient as the studies of cellular heterogeneity using single-cell data surges, and more and more cell types and markers are reported. We will extend ACT to other single-cell data, such as scATAC-seq, and upgrade it with more functionalities and new features in the near future. We anticipate ACT could do well in both identifying cell types and providing more convenience to the scientific community in assigning cell identities.

## Conclusions

In summary, we developed a knowledge-based resource, a corresponding method, and an intuitive graphical web interface for cell type annotation. And three case studies consistently showed that ACT assigned cell identities with high accuracy and precision and could provide more refined resolution and multiple levels cell annotation results, making the results comparable to expert manual annotation. We believe that ACT, as a powerful tool for annotating cell types, would be widely used and more efficient especially when the scale and volume of single-cell data continue to climb.

### Supplementary Information

**Additional file 1: Supplementary methods.** Comparing ACT with the CellMarker, PanglaoDB, clustermole, and MSigDB cell marker databases. **Fig. S1.** Unifying cell marker entries. **Fig. S3.** Effect of varying numbers of input DUGs on WISE prediction accuracy. **Fig. S4.** Frequency distribution of cell type markers in human blood. **Fig. S5.** The predicted results of WISE in the Human Liver dataset. **Fig. S6.** The performance of WISE. **Fig. S7.** An example of BatchACT. **Fig. S8.** Cell type annotation of the PBMC 3k dataset based on ACT. **Fig. S9.** Cell type annotation with ACT in basal cell carcinoma. **Table S3.** Result of cell type assignment for PMBC 3k scRNA-seq dataset based on ACT. **Table S4.** Manually assigned cell types based on the annotation results of ACT in BCC. **Table S5.** Manually cell type annotation based on the enrichment results of ACT in colon cancer.

**Additional file 2: Fig. S2. **Manual inspection and correction of originally incorrectly annotated clusters across five datasets.

**Additional file 3: Table S1. **Manual inspection and correction of originally incorrectly annotated clusters across five datasets. **Table S2.** The major-cell-type catalog used for mapping cell types to a unified level.

## Data Availability

The ACT web server is freely accessible and requires no registration. All single-cell datasets used in this study were obtained from public data repositories. Five datasets were used for the benchmarking analysis. The Tabula Sapiens dataset was downloaded from the Chan Zuckerberg Biohub (https://tabula-sapiens-portal.ds.czbiohub.org/) [13]. The human PBMC (10X PBMC 8k) dataset was obtained from the 10X Genomics website: https://support.10xgenomics.com/single-cell-gene-expression/datasets [23]. Human liver, mouse lung and mouse retina datasets were obtained from Gene Expression Omnibus (GEO), with accession numbers GSE124395 (https://www.ncbi.nlm.nih.gov/geo/query/acc.cgi?acc=GSE124395) [24], GSE124872 (https://www.ncbi.nlm.nih.gov/geo/query/acc.cgi?acc=GSE124872) [25] and GSE63473 (https://www.ncbi.nlm.nih.gov/geo/query/acc.cgi?acc=GSE63473) [26], respectively. The PBMC 3k dataset was obtained from the 10X Genomics website: https://support.10xgenomics.com/single-cell-gene-expression/datasets [23]. Data concerned with BCC was available at the GEO repository: GSE123814 (https://www.ncbi.nlm.nih.gov/geo/query/acc.cgi?acc=GSE123814) [29, 29]. The dataset of colon cancer was downloaded from Synapse under the accession code syn26844071 (https://www.synapse.org/#!Synapse:syn26844071/files/) [30]. For human and mouse, data for visualization of expression and metadata were retrieved from the single-cell atlases of the Tabula Sapiens [13], HCA [47, 48, 49], HCL [15], and MCA [14]. In addition, the dataset of mouse adipose tissue was available at Single Cell Portal: SCP1376 (https://singlecell.broadinstitute.org/single_cell/study/SCP1376/a-single-cell-atlas-of-human-and-mouse-white-adipose-tissue#study-download) [28]. The dataset of muscle-tendon junctions of mouse was available at GSE168153 (https://www.ncbi.nlm.nih.gov/geo/query/acc.cgi?acc=GSE168153) [45].

## Abbreviations

ACT: Annotation of cell types
scRNA-seq: Single-cell RNA sequencing
DEG: differentially expressed gene
WISE: Weighted and Integrated gene Set Enrichment
DUG: differentially upregulated gene
NES: Normalized Enrichment Score
PBMC: Peripheral blood mononuclear cell
BCC: Basal cell carcinoma
Th17: T-helper 17 cell
Tfh: T follicular helper cell
CTL: Cytotoxic T cell
Tem: Effector memory CD8-positive, alpha-beta T cell
DC: Dendritic cell
mregDC: Migratory dendritic cell
Treg: Regulatory T cell
CD8_act_T_cells: CD8+ activated T cell
CD8_ex_T_cells: CD8+ exhausted T cell
pDC: Plasmacytoid dendritic cell
CAF: Cancer-associated fibroblast
Tcell_prolif: Proliferating T cell
CD4_Tn: Naive thymus-derived CD4-positive, alpha-beta T cell
CD8_Tem: Effector memory CD8-positive alpha, beta T cell
NK: Natural killer cell
FO_B: Follicular B cell
Endo: Endothelial cell
Endo_lymphatic: Lymphatic endothelial cell
Fibro: Fibroblast
Myof: Myofibroblast cell
Epi_colon: Colon epithelial cell
Goblet_T: Malignant goblet cell
Goblet_N: Normal goblet cell
McDC: Monocyte conventional dendritic cell
GEO: Gene Expression Omnibus
