# Supplementary material for: Annotation of cell types (ACT): a convenient web server for cell type annotation
Source: Genome Med. 2023 Nov 3;15:91. doi: 10.1186/s13073-023-01249-5 (PMC10623726; doi:10.1186/s13073-023-01249-5)
Supplement: Supplementary file 1 — Additional file 1: Supplementary methods. Comparing ACT with the CellMarker, PanglaoDB, clustermole, and MSigDB cell marker databases. Fig. S1. Unifying cell marker entries. Fig. S3. Effect of varying numbers of input DUGs on WISE prediction accuracy. Fig. S4. Frequency distribution of cell type markers in human blood. Fig. S5. The predicted results of WISE in the Human Liver dataset. Fig. S6. The performance of WISE. Fig. S7. An example of BatchACT. Fig. S8. Cell type annotation of the PBMC 3k dataset based on ACT. Fig. S9. Cell type annotation with ACT in basal cell carcinoma. Table S3. Result of cell type assignment for PMBC 3k scRNA-seq dataset based on ACT. Table S4. Manually assigned cell types based on the annotation results of ACT in BCC. Table S5. Manually cell type annotation based on the enrichment results of ACT in colon cancer. [file 13073_2023_1249_MOESM1_ESM.docx]

**Supplementary materials**

**Supplementary methods**

Comparing ACT with the CellMarker, PanglaoDB, clustermole, and MSigDB cell marker databases

**Supplementary figures**

Fig. S1 Unifying cell marker entries.

Fig. S2 Manual inspection and correction of originally incorrectly annotated clusters across five datasets. (additional file).

Fig. S3 Effect of varying numbers of input DUGs on WISE prediction accuracy.

Fig. S4 Frequency distribution of cell type markers in human blood.

Fig. S5 The predicted results of WISE in the Human Liver dataset.

Fig. S6 The performance of WISE.

Fig. S7 An example of BatchACT.

Fig. S8 Cell type annotation of the PBMC 3k dataset based on ACT.

Fig. S9 Cell type annotation with ACT in basal cell carcinoma.

**Supplementary tables**

Table S1. Manual inspection and correction of originally incorrectly annotated clusters across five datasets (additional file).

Table S2. The major-cell-type catalog used for mapping cell types to a unified level (additional file).

Table S3. Result of cell type assignment for PMBC 3k scRNA-seq dataset based on ACT.

Table S4. Manually assigned cell types based on the annotation results of ACT in BCC.

Table S5. Manually cell type annotation based on the enrichment results of ACT in colon cancer.

**Supplementary methods**

**Comparing ACT with the CellMarker, PanglaoDB, clustermole, and MSigDB cell marker databases**

We compared ACT with cell marker databases, CellMarker [1], PanglaoDB [2], clustermole [3], and MSigDB [4], regarding the numbers of collected tissue types, cell types, and literatures.

We initially compared ACT with CellMarker. ACT encompassed a total of 282 tissues and 806 cell types in humans, along with 240 tissues and 867 cell types in mice. In contrast, CellMarker included 158 tissues and 467 cell types for humans and 81 tissues and 389 cell types for mice. Our observation revealed that ACT provided a more extensive range of tissues and cell types. For instance, when considering brain tissue, ACT included 95 human cell types and 118 mouse cell types, whereas CellMarker contained only 43 and 54 cell types for humans and mice, respectively. Notably, certain cell types such as Pericytes, Radial glial cells in humans, and Neuronal stem cells, Fibroblasts in mice were absent in CellMarker.

Similarly, we found that ACT encompassed more tissues and cell types compared to PanglaoDB (latest version as of March 27, 2020). PanglaoDB shared 29 tissues and 178 cell types between humans and mice. Using the example of brain tissue again, PanglaoDB included 35 cell types for both humans and mice. However, specific cell types like Oligodendrocyte precursor cells in humans and Glutamatergic neurons, Neutrophils in mice were not present in PanglaoDB.

The clustermole database (v1.1.0.9000) had fewer tissue types than ACT, with 81 human tissues and 53 mouse tissues in clustermole. A direct comparison of cell types was not feasible due to inconsistent nomenclature.

Furthermore, when compared to the MSigDB resource (v2023.1, C8 and M8 collections, cell type signature), ACT contained a more extensive collection of cell marker entries, including 13,700 entries for humans and 10,126 entries for mice. In MSigDB, there were only 830 entries for humans and 232 for mice (MSigDB didn’t clearly provide tissue and cell types).

Additionally, we extracted literature references included in ACT and other cell marker resources separately. ACT contained references from over 3,600 articles, with 1,538 articles exclusively provided by ACT (PanglaoDB and clustermole did not include article information in their released files).

In summary, our comparative analysis demonstrates that ACT offers a broader coverage of tissue and cell types, thus providing a comprehensive resource for cell marker information.

**References**

1. Zhang X, Lan Y, Xu J, Quan F, Zhao E, Deng C, Luo T, Xu L, Liao G, Yan M, et al: **CellMarker: a manually curated resource of cell markers in human and mouse.** *Nucleic Acids Res* 2019, **47:**D721-D728.

2. Franzen O, Gan LM, Bjorkegren JLM: **PanglaoDB: a web server for exploration of mouse and human single-cell RNA sequencing data.** *Database (Oxford)* 2019, **2019**.

3. Dolgalev I: **clustermole: Unbiased single-cell transcriptomic data cell type identification.** 2021.

4. Liberzon A, Birger C, Thorvaldsdottir H, Ghandi M, Mesirov JP, Tamayo P: **The Molecular Signatures Database (MSigDB) hallmark gene set collection.** *Cell Syst* 2015, **1:**417-425.

**Supplementary figures**


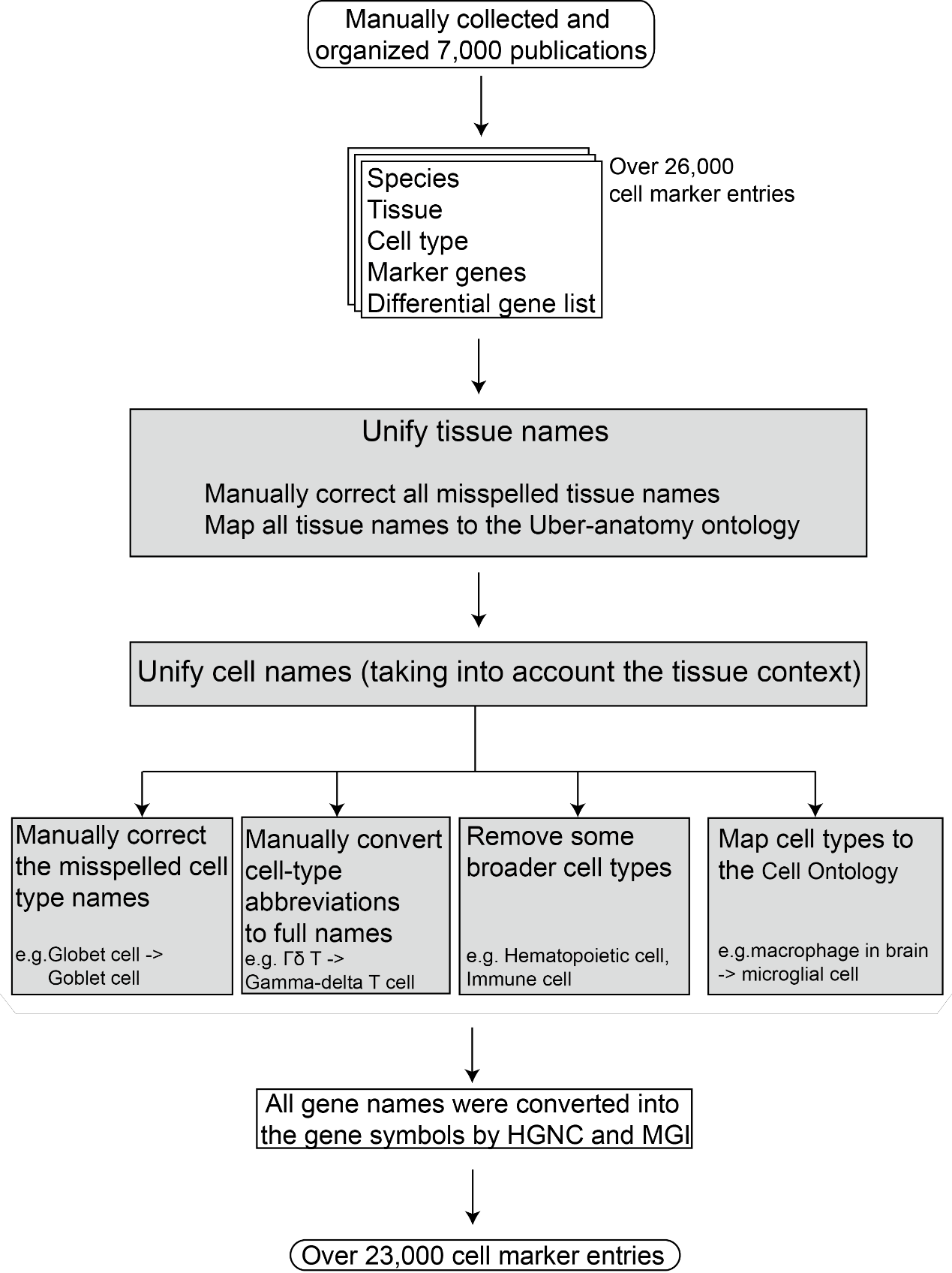


**Fig. S1** Unifying cell marker entries.


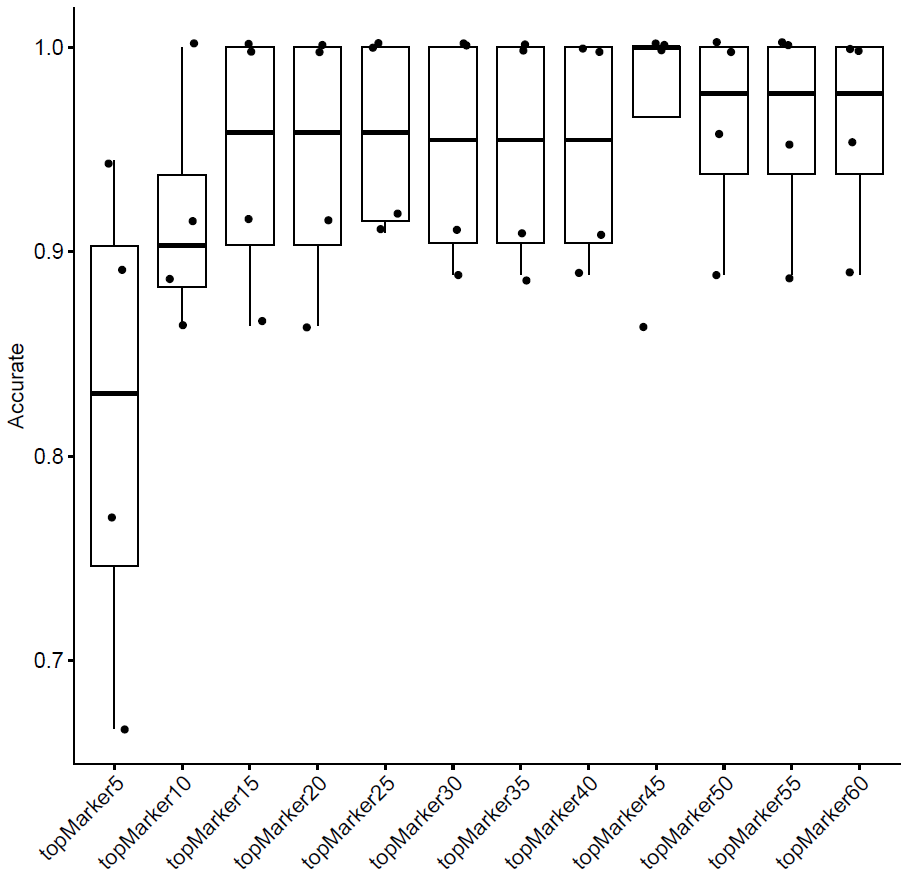


**Fig. S3** The prediction accuracy of WISE varies when using the top 5, 10, ..., and 60 DUGs from each cluster as input across 4 datasets (human liver, human PBMC, mouse lung, and mouse retina).


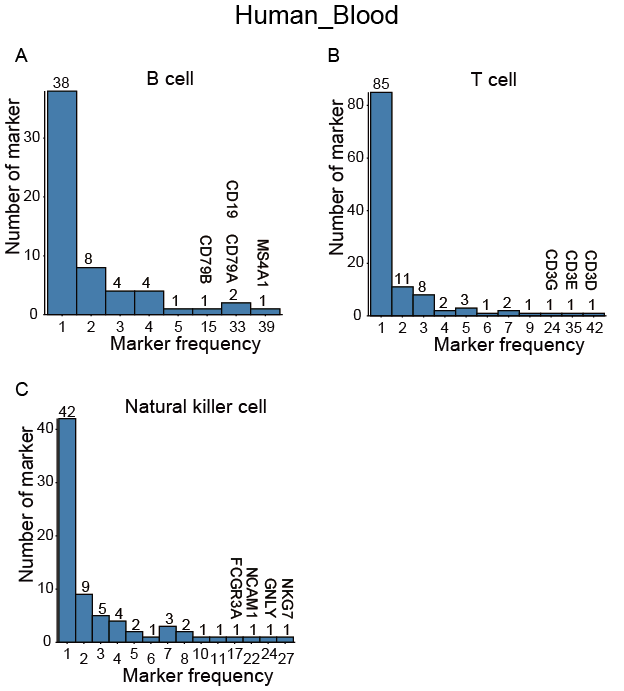


**Fig. S4** Frequency distribution of cell type markers in human blood. Distribution of usage frequency for B cell markers (**A**), T cell markers (**B**), and NK cell markers (**C**).


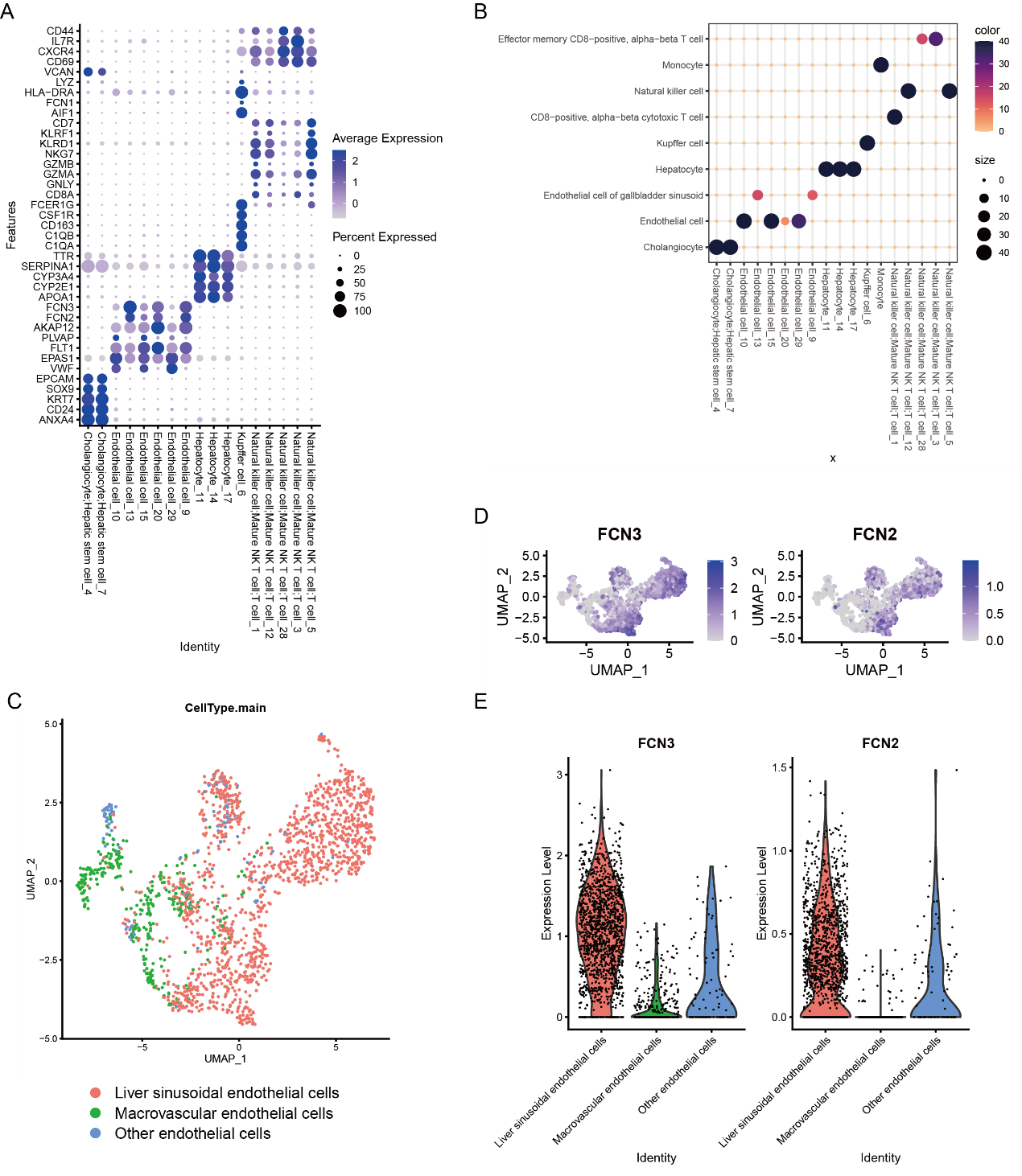


**Fig.** S5 The predicted results of WISE in the Human Liver dataset. (**A**) Expression of canonical markers for each cluster in the Human Liver dataset. (**B**) The top one result that WISE predicted for each cluster. The color and size of the dots are displayed based on the enrichment significance. (**C**) Dimensionality reduction plot of endothelial cell subtypes in the Human Liver dataset. (**D**) Expression of canonical markers in liver sinusoidal endothelial cells. (**E**) Violin plot depicting the expression of canonical markers in Liver Sinusoidal Endothelial Cells.


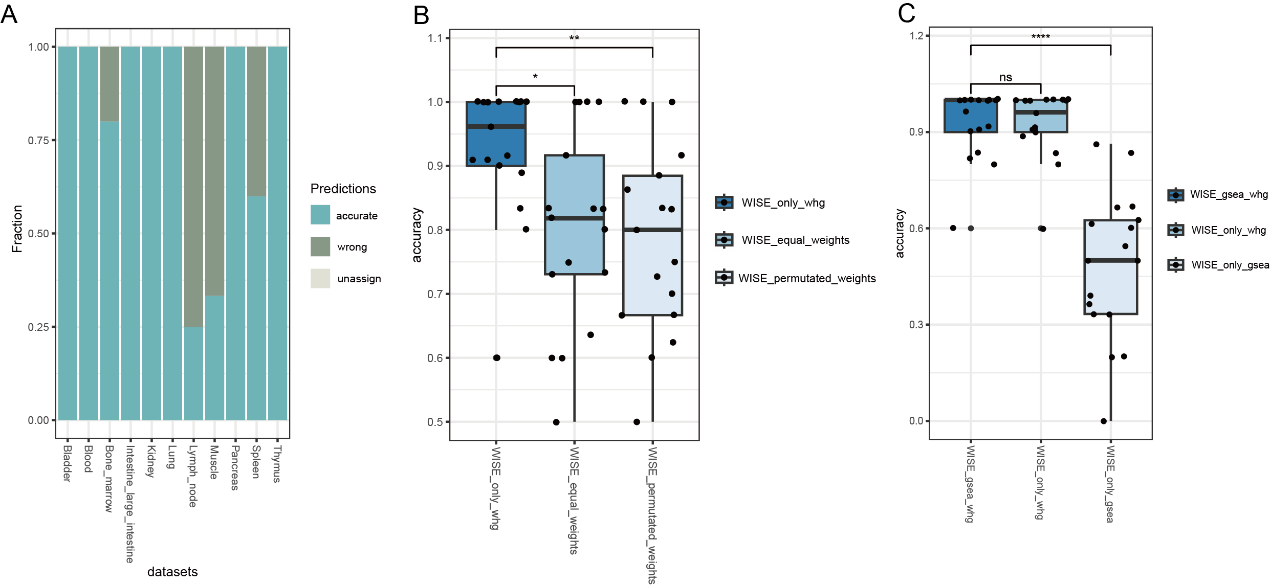


**Fig. S6** The performance of WISE. (**A**) The fraction of accurately predicted clusters, incorrectly predicted clusters, and unassigned clusters in the predictions made by WISE in Tabula Sapiens dataset (Smart-seq2). (**B**) The performance of WISE with weights (WISE_only_whg), with equal weights of 1 (WISE_equal_weights), and with permutated weights (WISE_permutated_weights). (**C**) The performance of WISE combined with GSEA (WISE_gsea_whg), without GSEA (WISE_only_whg), and only with GSEA (WISE_only_gsea).


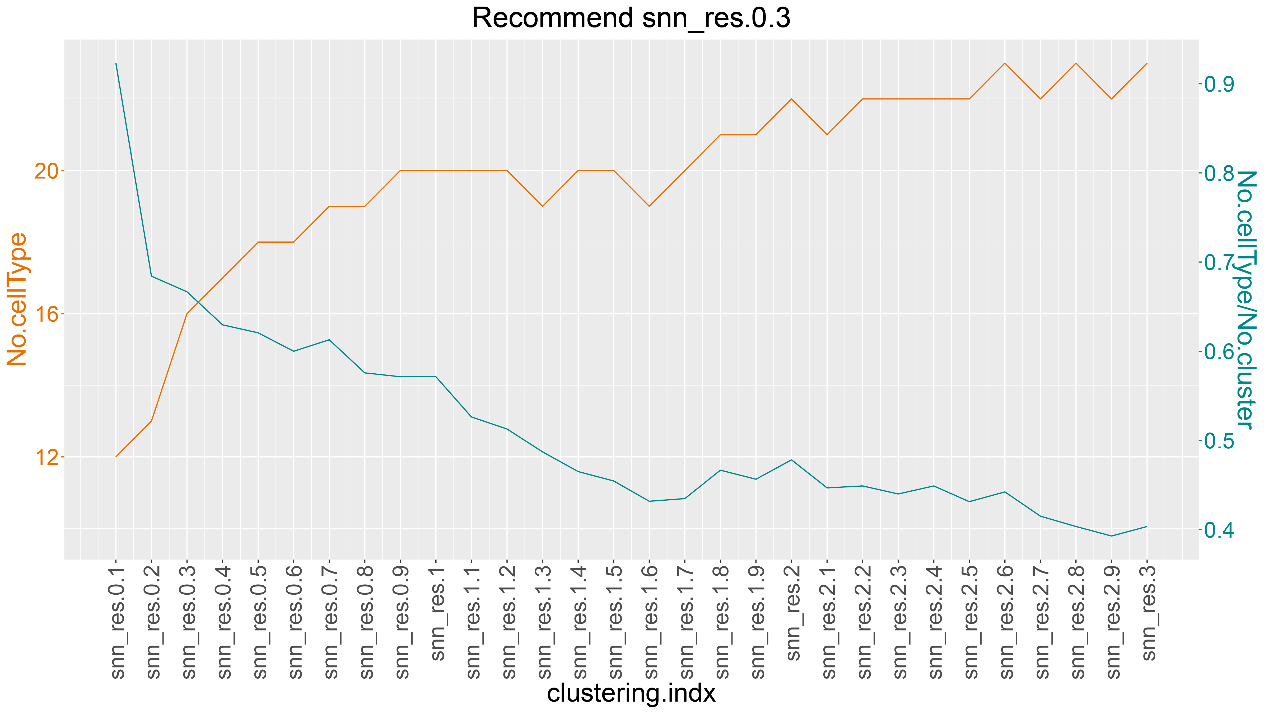


**Fig. S7** BatchACT results across 30 resolution values (0.1-3) in the Tablus_Human_Lung dataset. No.cellType: the count of unique cell types annotated by BatchACT; No.cellType/No.cluster: ratio of No.cellType to the total number of clusters.


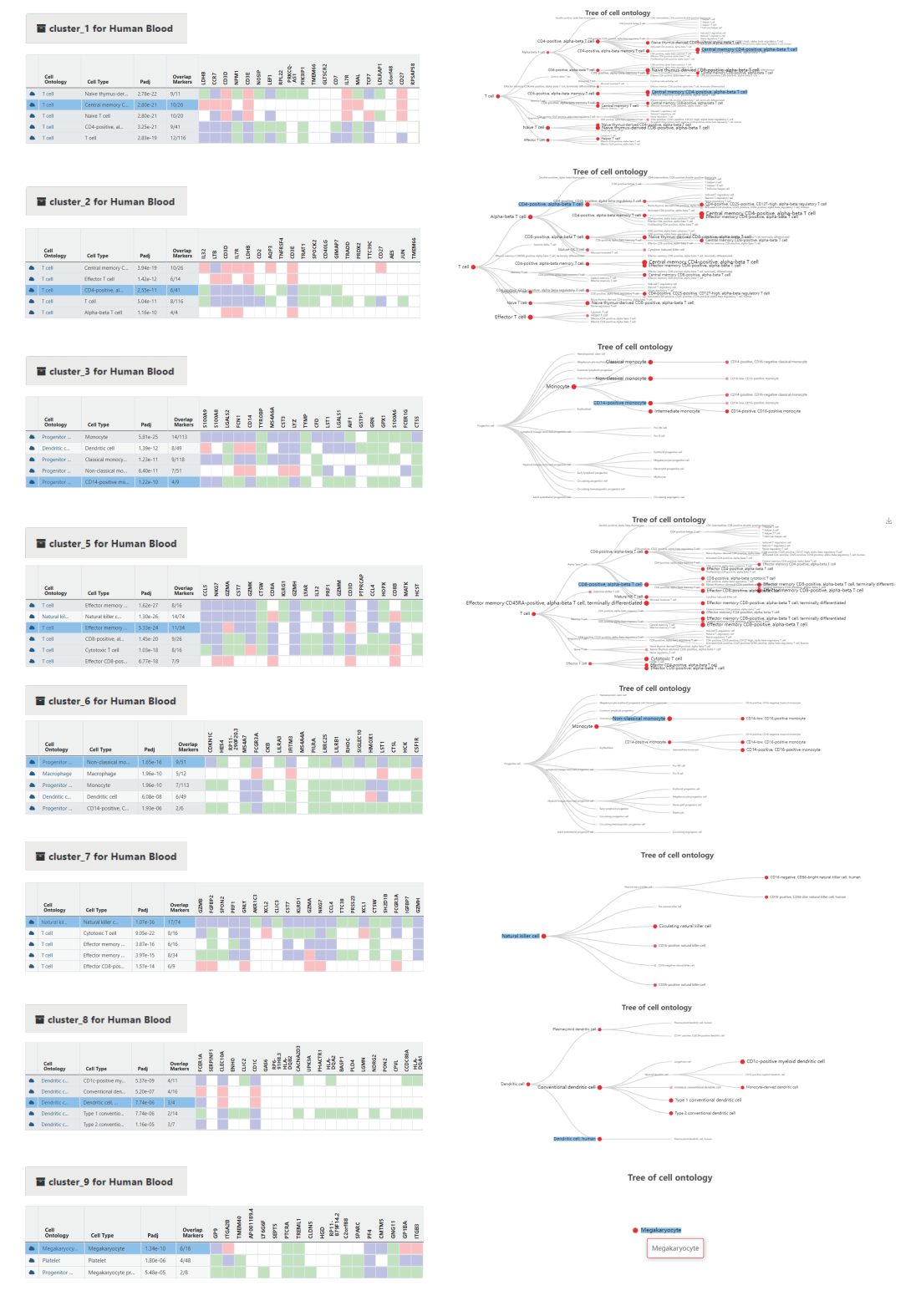


**Fig. S8** Cell type annotation of the PBMC 3k dataset based on ACT. Main summary tables and interactive hierarchy maps are displayed.


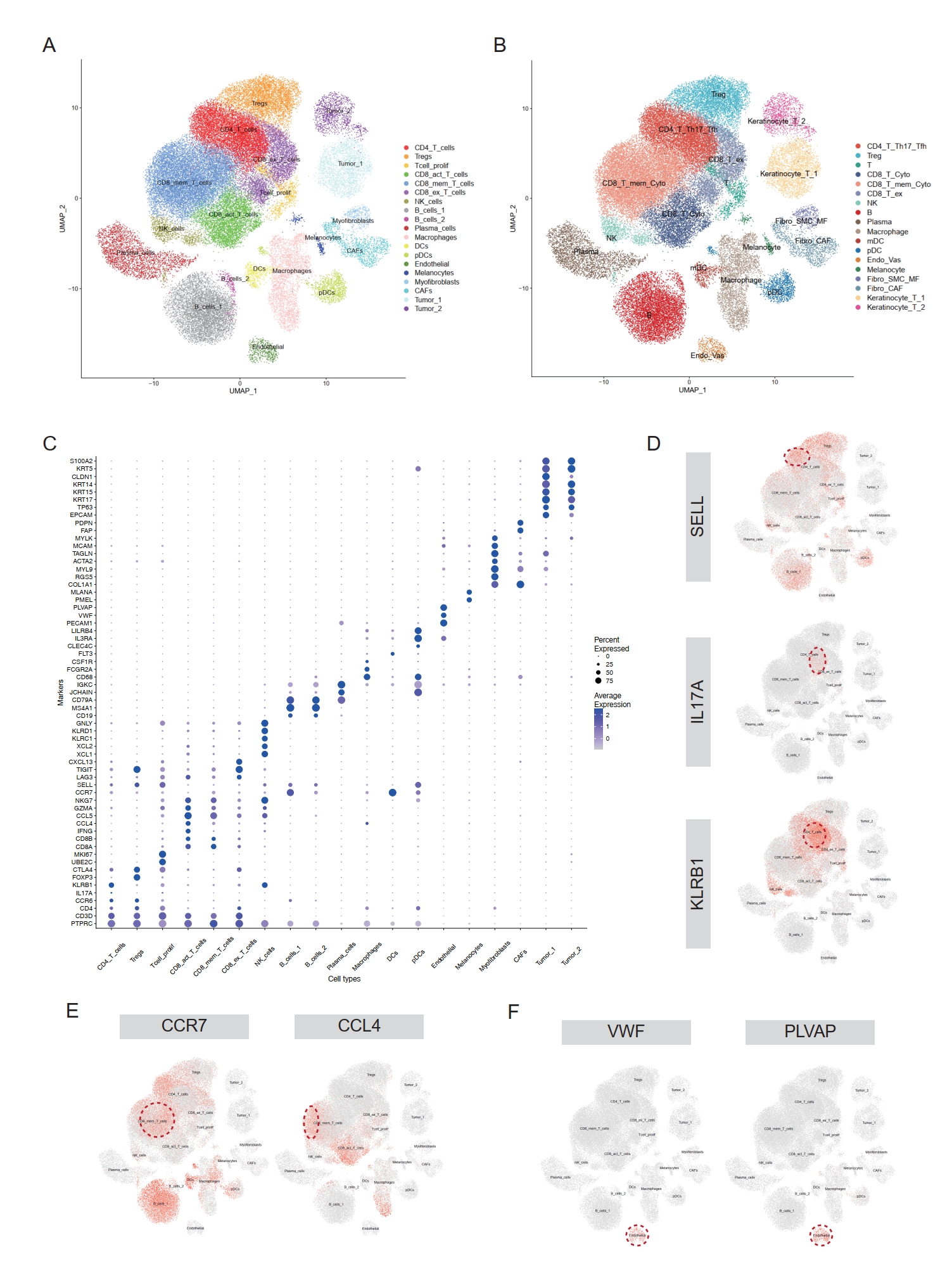


**Fig. S9** Cell type annotation with ACT in basal cell carcinoma. (**A**) UMAP projection of original cell labels. (**B**) The same UMAP plot as (**A**) but colored by our annotation result based on ACT. (**C**) Dot plot shows pre-defined cell types and the expression of marker genes. (**D**) Another three subtypes of T cells in CD4+ T cells. (**E**) Two phenotypes in CD8+ memory T cells. (**F**) Feature plots depicting expression of canonical markers in vascular endothelial cells.

**Supplementary tables**

Table S3. Result of cell type assignment for PMBC 3k scRNA-seq dataset based on ACT.

| **Cluster** | **Original cell labels** | **# of cells** | **Most significant annotations (n=2)** | **Manually assigned cell types based on ACT** |
| --- | --- | --- | --- | --- |
| 1 | Naive CD4 T | 697 | Naïve T cell  T cell | Naive T cell |
| 2 | Memory CD4 T | 483 | CD4-positive, alpha-beta memory T cell  T cell | Central memory CD4-positive, alpha-beta T cell |
| 3 | CD14+ Mono | 480 | Monocyte  Macrophage | Monocyte |
| 4 | B | 344 | B cell  Granulocyte | B cell |
| 5 | CD8 T | 271 | Natural killer cell  Effector CD8-positive, alpha-beta T cell | Effector CD8-positive, alpha-beta T cell |
| 6 | FCGR3A+ Mono | 162 | Monocyte  Non-classical monocyte | Non-classical monocyte |
| 7 | NK | 155 | Natural killer cell  Effector CD8-positive, alpha-beta T cell | Natural killer cell |
| 8 | DC | 32 | Dendritic cell  Monocyte-derived dendritic cell | Dendritic cell |
| 9 | Platelet | 14 | Megakaryocyte  Platelet | Megakaryocyte |

Note: cell types in red are annotation results that completely match the original cell labels, while the green ones indicate very similar results.

Table S4. Manually assigned cell types based on the annotation results of ACT in BCC.

| **Original cluster** | **# of cells** | **ACT_cellType** | **Markers** |
| --- | --- | --- | --- |
| CD4_T_cells | 7765 | T-helper 17 cell/T-helper 22 cell/T follicular helper cell/CD4-positive, alpha-beta T cell | PTPRC, CD3D, CD4, CCR6, IL17A, KLRB1, CD40LG |
| Tregs | 4540 | Regulatory T cell | FOXP3, CTLA4 |
| Tcell_prolif | 857 | T cell | UBE2C, MKI67 |
| CD8_act_T_cells | 4198 | Cytotoxic T cell | IFNG, CCL4, CCL5, GZMA, NKG7 |
| CD8_mem_T_cells | 12010 | Cytotoxic T cell/Effector memory CD8-positive, alpha-beta T cell | CD8A, CD8B, CCR7, SELL, IL7R |
| CD8_ex_T_cells | 3736 | Exhausted T cell | LAG3, TIGIT, CXCL13 |
| NK_cells | 1041 | Natural killer cell | XCL1, XCL2, KLRC1, KLRD1, GNLY |
| B_cells_1 | 5829 | B cell | CD19, MS4A1 |
| B_cells_2 | 194 | B cell | CD79A |
| Plasma_cells | 2647 | Plasma cell | JCHAIN, IGKC |
| Macrophages | 3093 | Macrophage | CD68, FCGR2A, CSF1R |
| DCs | 469 | Migratory dendritic cell | FLT3, FCER1A, CD1C, CCR7, FSCN1 |
| pDCs | 979 | Plasmacytoid dendritic cells | CLEC4C, IL3RA, LILRB4 |
| Endothelial | 471 | Endothelial cell | PECAM1, VWF, PLVAP |
| Melanocytes | 206 | Melanocyte | PMEL, MLANA |
| Myofibroblasts | 378 | Skin fibroblast/Smooth muscle cell/Myofibroblast cell | COL1A1, RGS5, MYL9, ACTA2, TAGLN, MCAM, MYLK |
| CAFs | 1066 | Skin fibroblast/Cancer-associated fibroblast | FAP, PDPN |
| Tumor_1 | 2462 | Keratinocyte | EPCAM, TP63 |
| Tumor_2 | 1089 | Keratinocyte | KRT17, KRT15, KRT14, CLDN1, KRT5, S100A2 |

Table S5. Manually cell type annotation based on the enrichment results of ACT in colon cancer.

| **Cluster** | **# of cells** | **Original label** | **Ground_truth label** | **ACT-based annotation** |
| --- | --- | --- | --- | --- |
| 0 | 29830 | T_NK | Naive thymus-derived CD4-positive, alpha-beta T cell | Naive thymus-derived CD4-positive, alpha-beta T cell |
| 43 | 137 | T_NK | Naive thymus-derived CD4-positive, alpha-beta T cell | Naive thymus-derived CD4-positive, alpha-beta T cell |
| 3 | 13792 | T_NK | T-helper 17 cell | T-helper 17 cell |
| 13 | 6272 | T_NK | Regulatory T cell | Regulatory T cell |
| 1 | 17149 | T_NK | Effector memory CD8-positive, alpha-beta T cell | Effector memory CD8-positive, alpha-beta T cell |
| 6 | 11161 | T_NK | Effector memory CD8-positive, alpha-beta T cell | Effector memory CD8-positive T cell |
| 21 | 2979 | T_NK | Effector memory CD8-positive, alpha-beta T cell | Effector memory CD8-positive, alpha-beta T cell |
| 30 | 1035 | T_NK | Cytotoxic T cell | Cytotoxic T cell |
| 35 | 767 | T_NK | Natural killer cell | Natural killer cell |
| 2 | 14221 | B | Follicular B cell | Follicular B cell |
| 18 | 4734 | B | Follicular B cell | Follicular B cell |
| 5 | 11858 | PlasmaB | Plasma cell | Plasma cell |
| 7 | 10302 | PlasmaB | Plasma cell | Plasma cell |
| 8 | 9863 | PlasmaB | Plasma cell | Plasma cell |
| 39 | 374 | PlasmaB | Plasma cell | Plasma cell |
| 44 | 128 | PlasmaB | Plasma cell | Plasma cell |
| 4 | 13703 | McDC | Colon macrophage | Colon macrophage |
| 42 | 225 | McDC | Colon macrophage | Colon macrophage |
| 9 | 9049 | McDC | Granulocyte | Granulocyte |
| 27 | 1871 | McDC | Migratory dendritic cell | Migratory dendritic cell |
| 33 | 988 | Neutrophils | Neutrophil | Neutrophil |
| 28 | 1744 | Mast | Mast cell | Mast cell |
| 23 | 2835 | Entericglial | Glial cell | Glial cell |
| 10 | 7446 | Endothelial | Endothelial cell | Endothelial cell |
| 41 | 309 | Endothelial | Endothelial cell | Endothelial cell |
| 38 | 624 | Endothelial | Endothelial cell of lymphatic vessel | Endothelial cell of lymphatic vessel |
| 12 | 7311 | Fibroblast | Fibroblast | Fibroblast |
| 15 | 5736 | Fibroblast | Fibroblast | Fibroblast |
| 16 | 5179 | Fibroblast | Fibroblast | Fibroblast |
| 24 | 2638 | Fibroblast | Fibroblast | Fibroblast |
| 26 | 2356 | Fibroblast | Fibroblast | Fibroblast |
| 32 | 1001 | Fibroblast | Fibroblast | Fibroblast |
| 34 | 910 | Fibroblast | Fibroblast | Fibroblast |
| 40 | 360 | Fibroblast | Fibroblast | Fibroblast |
| 14 | 6054 | Fibroblast | Myofibroblast cell | Myofibroblast cell |
| 11 | 7312 | Epithelial | Colon epithelial cell | Colon epithelial cell |
| 17 | 4881 | Epithelial | Colon epithelial cell | Colon epithelial cell |
| 29 | 1512 | Epithelial | Colon epithelial cell | Colon epithelial cell |
| 31 | 1005 | Epithelial | Colon epithelial cell | Colon epithelial cell |
| 36 | 764 | Epithelial | Colon epithelial cell | Colon epithelial cell |
| 19 | 4417 | Epithelial | Colon goblet cell | Colon goblet cell |
| 20 | 4406 | Epithelial | Colon goblet cell | Colon goblet cell |
| 22 | 2925 | Epithelial | Colon goblet cell | Colon goblet cell |
| 25 | 2463 | Epithelial | Colon goblet cell | Colon goblet cell |
| 37 | 706 | Epithelial | Colon goblet cell | Colon goblet cell |
