# Supplementary material for: Annotation of cell types (ACT): a convenient web server for cell type annotation
Source: Genome Med. 2023 Nov 3;15:91. doi: 10.1186/s13073-023-01249-5 (PMC10623726; doi:10.1186/s13073-023-01249-5)

**Fig. S2** Manual inspection and correction of originally incorrectly annotated clusters across five datasets.

In Tabula Sapiens Bladder, the cluster labeled as "Myofibroblast cell" was found to be a mixture of "Myofibroblast cell" and "Fibroblast" (A). This cluster was determined to represent a mixture of both Fibroblast and Myofibroblast cell, exhibiting high expression of the Myofibroblast cell marker, ACTA2, MYH11, TAGLN, and MYL9 (B and C), as well as elevated expression of Fibroblast markers like PDGFRA, COL1A1, LUM, and DCN (D and E).


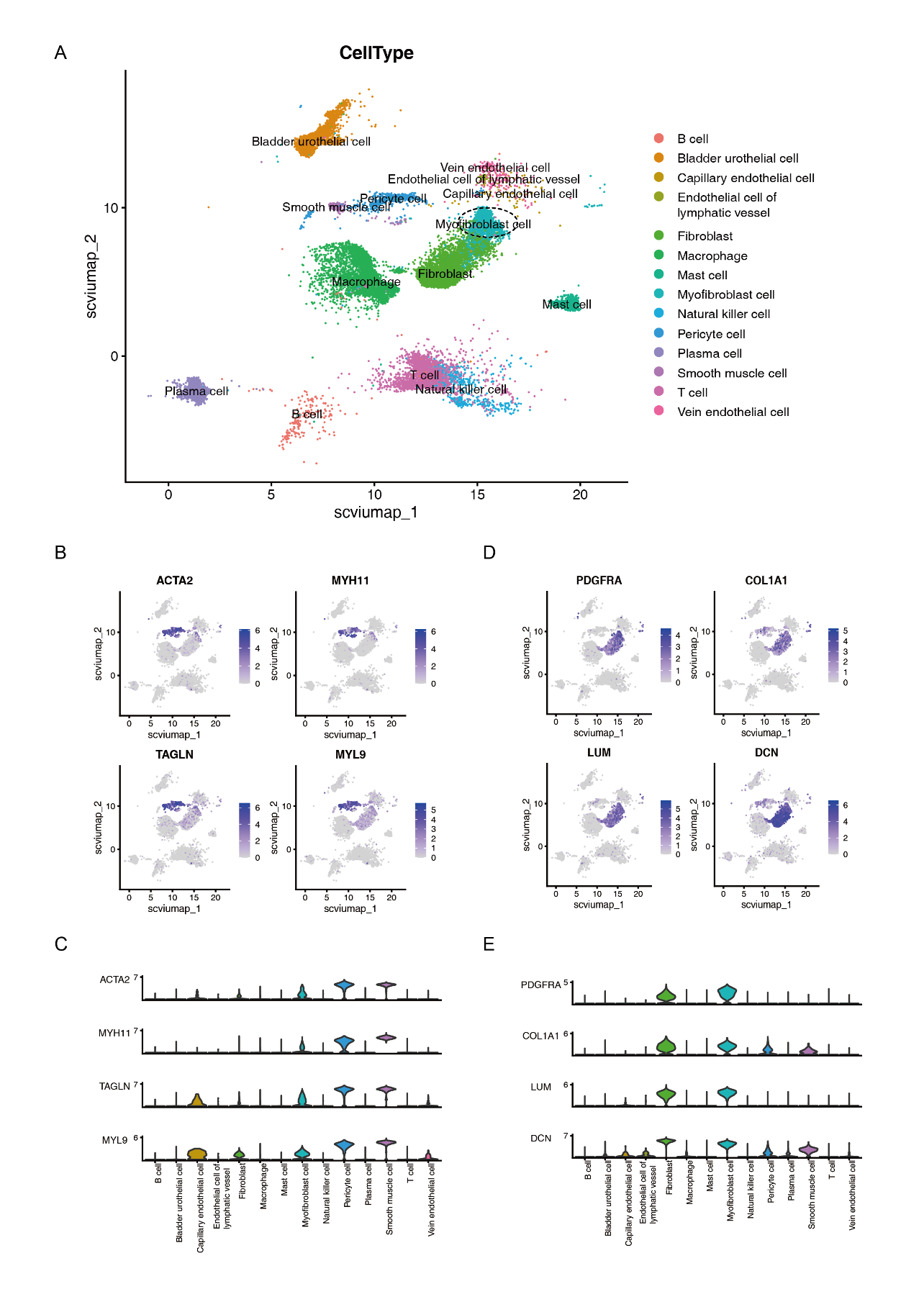


In Tabula Sapiens Bladder, the cluster labeled as "Natural killer cell" was found to be a mixture of "Natural killer cell" and " Mature NK T cell" (A). This cluster was determined to represent a mixture of both Mature NK T cell and Natural killer cell, exhibiting high expression of the Natural killer cell marker, NKG7, GNLY, KLRD1, and NCAM1 (B and C), as well as elevated expression of markers Mature NK T cell like CD3D and CD8A (D and E).


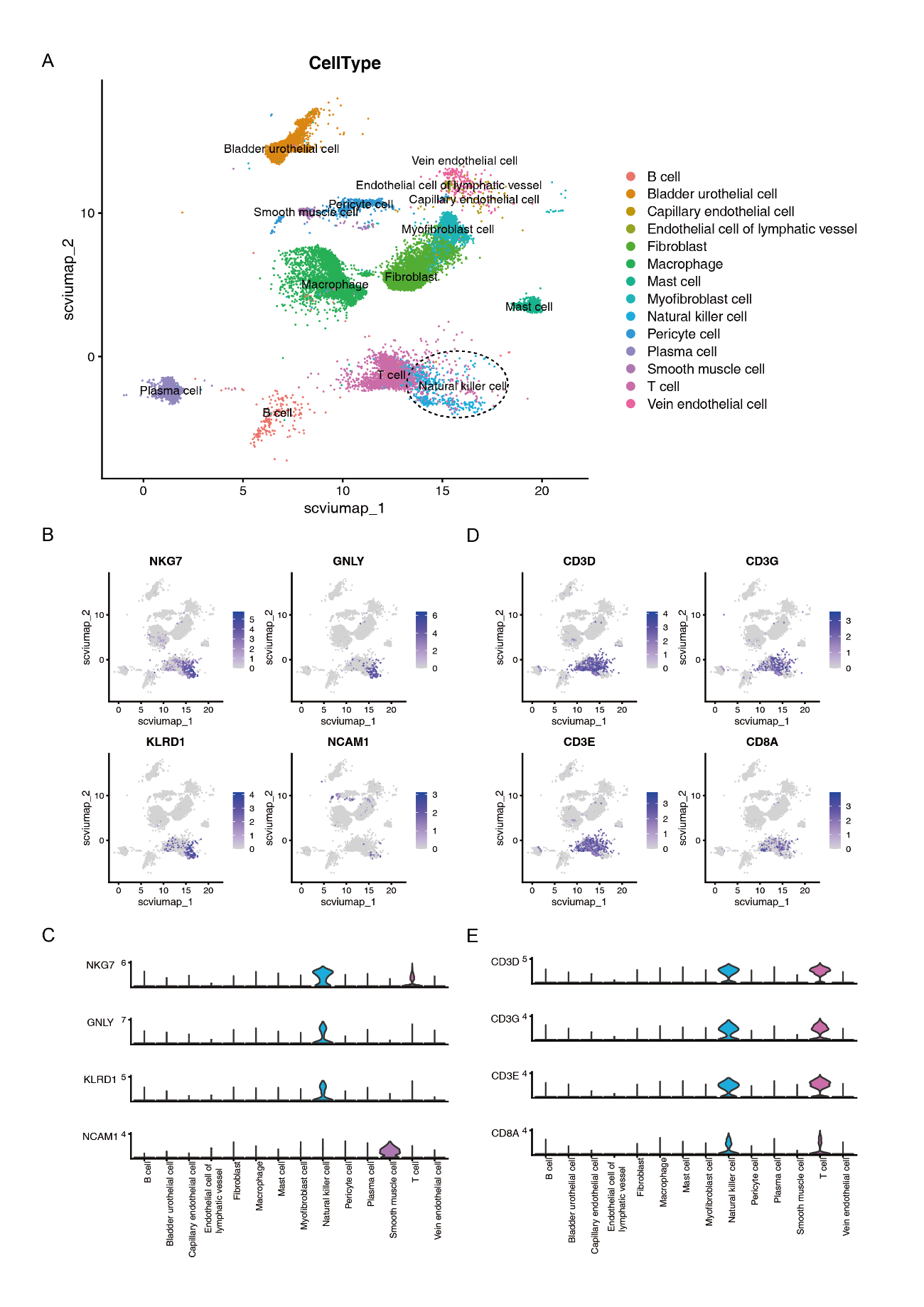


In Tabula Sapiens Bone marrow, the cluster labeled as "CD4-positive, alpha-beta T cell" may have been inaccurately annotated previously (A), as it exhibited high expression of the Naive T cell markers, such as SELL, TCF7, CCR7, and LEF1 (D and E), while showing low expression of markers for CD4-positive, alpha-beta T cells, including CD4, IL7R, CD3E, and CD3D (B and C).


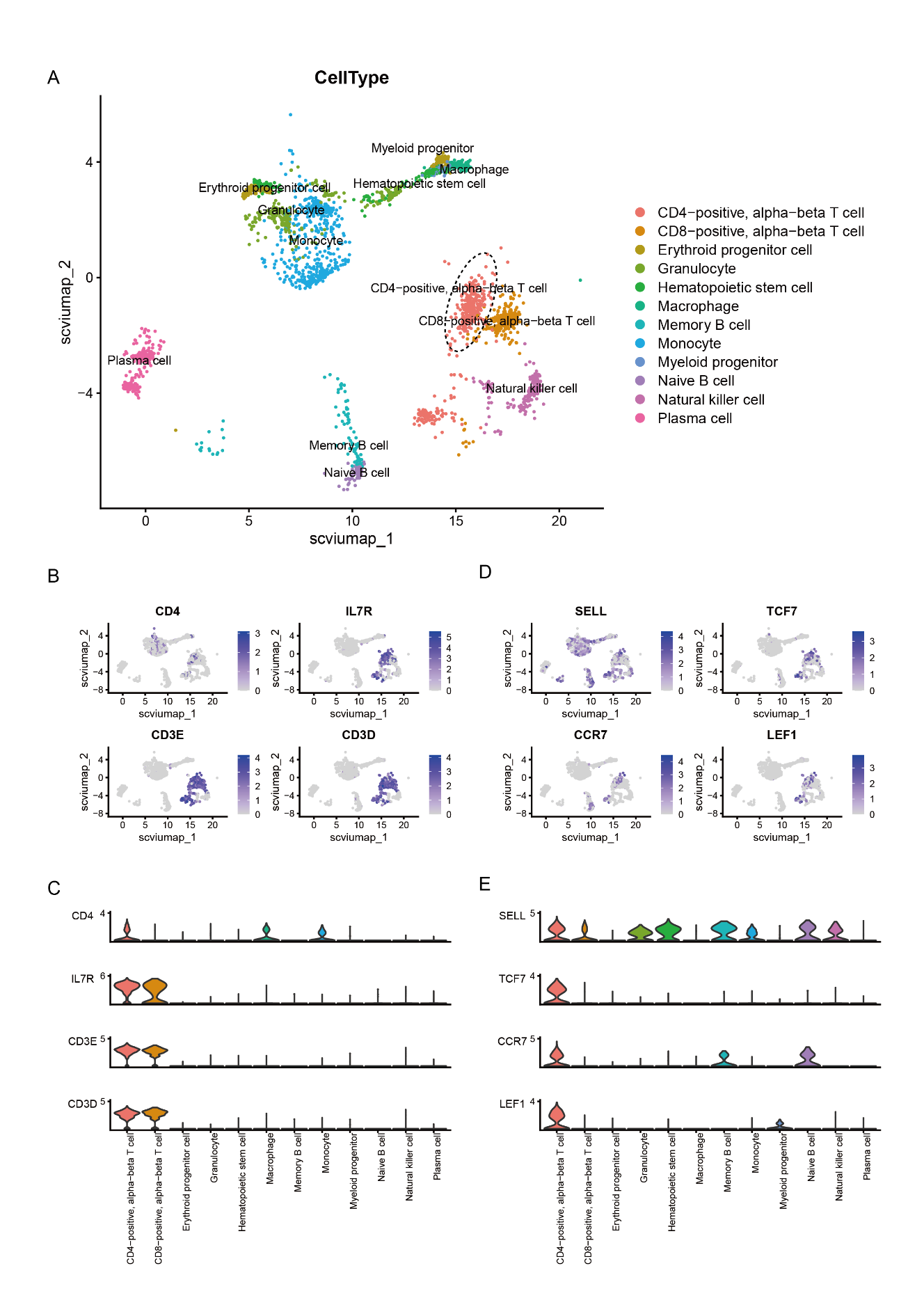


In Tabula Sapiens Kidney, the cluster labeled as "Macrophage" was found to be a mixture of "Macrophage" and "Monocyte" (A). This cluster was determined to represent a mixture of both Monocyte cell and Macrophage, exhibiting high expression of the Macrophage marker, C1QA, 1QB, and D3D (B and C), as well as elevated expression of Monocyte markers like FCN1 and VCAN (D and E).


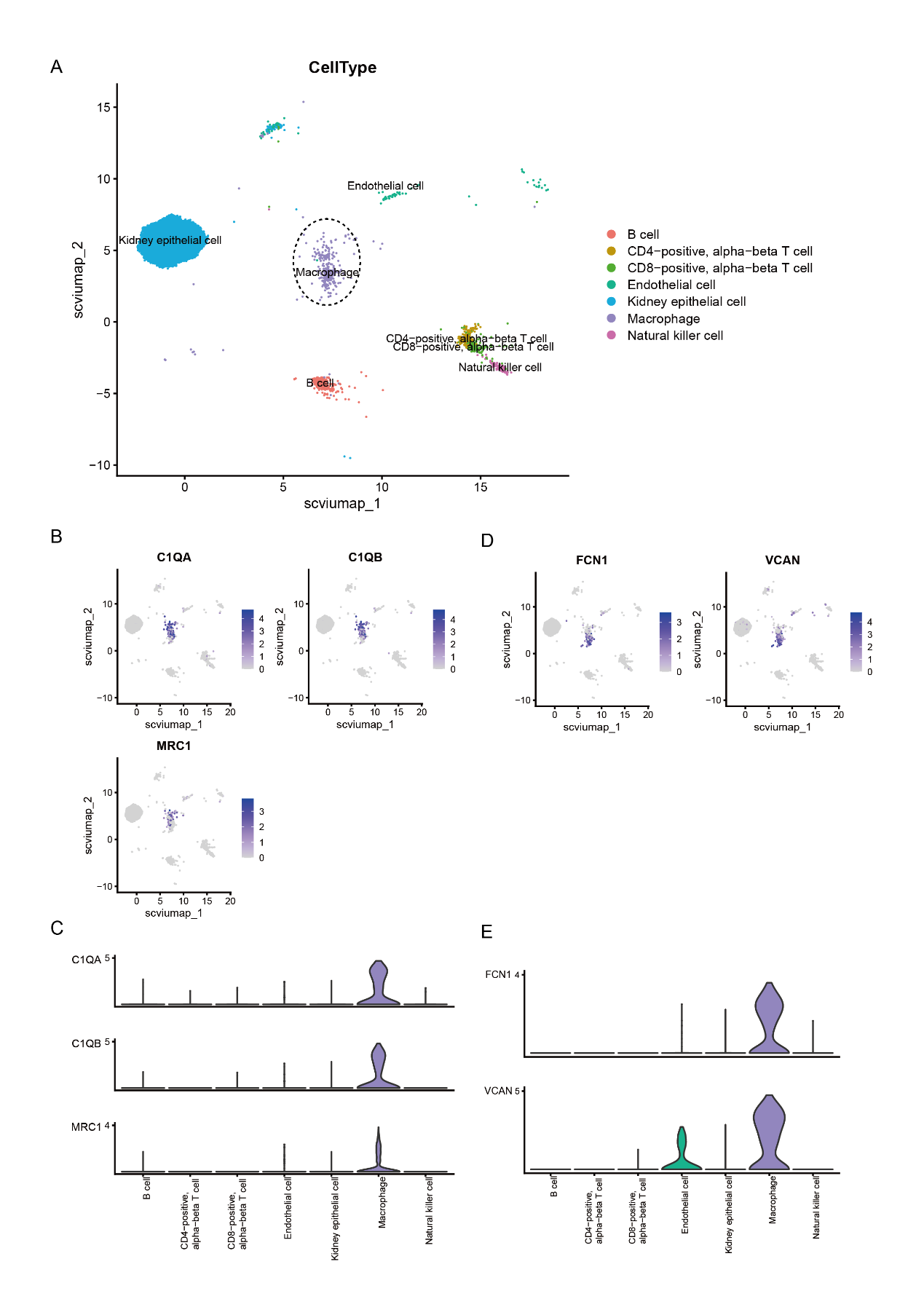


In Tabula Sapiens Lung, the cluster labeled as "Basophil" may have been inaccurately annotated previously (A), as it exhibited high expression of the Mast cell markers, such as TPSB2, TPSAB1, CPA3, and MS4A2 (D and E), while showing low expression of markers for Basophil, including CD63 and ENPP3 (C and B).


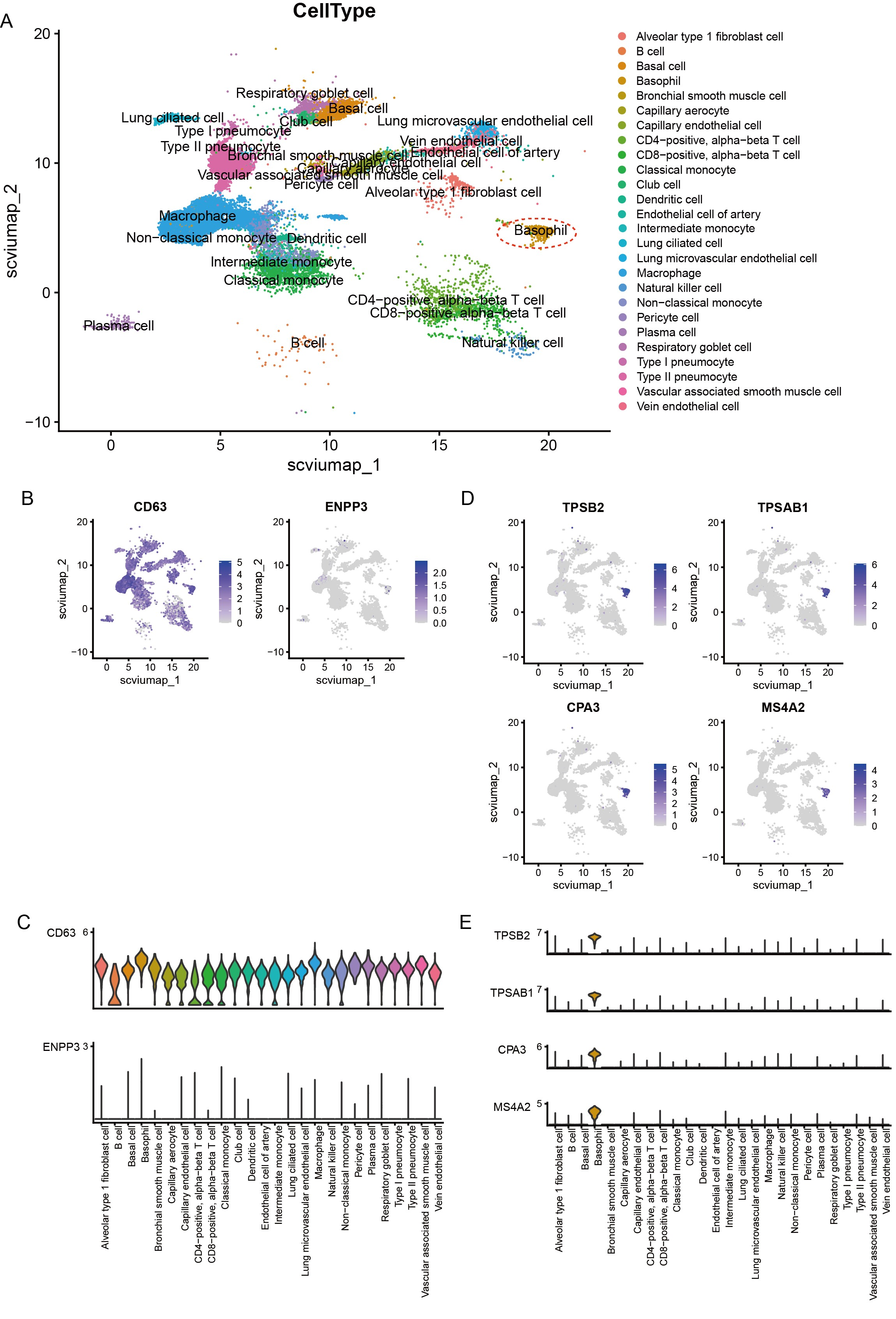


In Tabula Sapiens Lung, the cluster labeled as "Respiratory goblet cell" may have been inaccurately annotated previously (A), as it exhibited high expression of the Club cell markers, such as SCGB3A1, SCGB1A1, BPIFB1, and CYP2F1 (D and E), while showing low expression of markers for Respiratory goblet cell, including MUC5AC, TFF3, MUC2, and SPDEF (C and B).


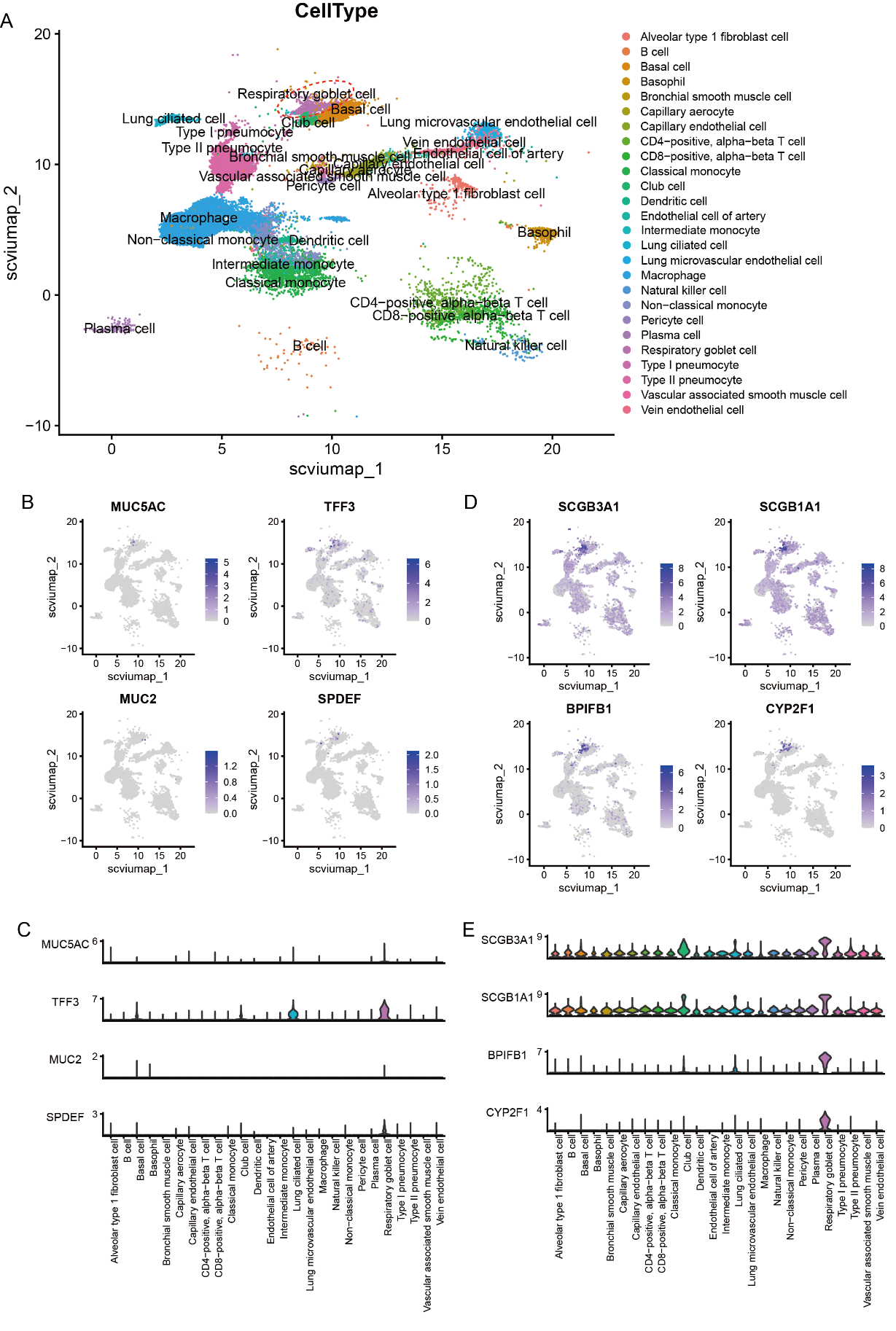


In Tabula Sapiens Lung Trachea, the cluster labeled as "Goblet cell" may have been inaccurately annotated previously (A), as it exhibited high expression of the Club cell markers, such as SCGB3A1, and MUC5B (D and E), while showing low expression of markers for Goblet cell, including MUC5AC, TFF3, MUC2, and SPDEF (C and B).


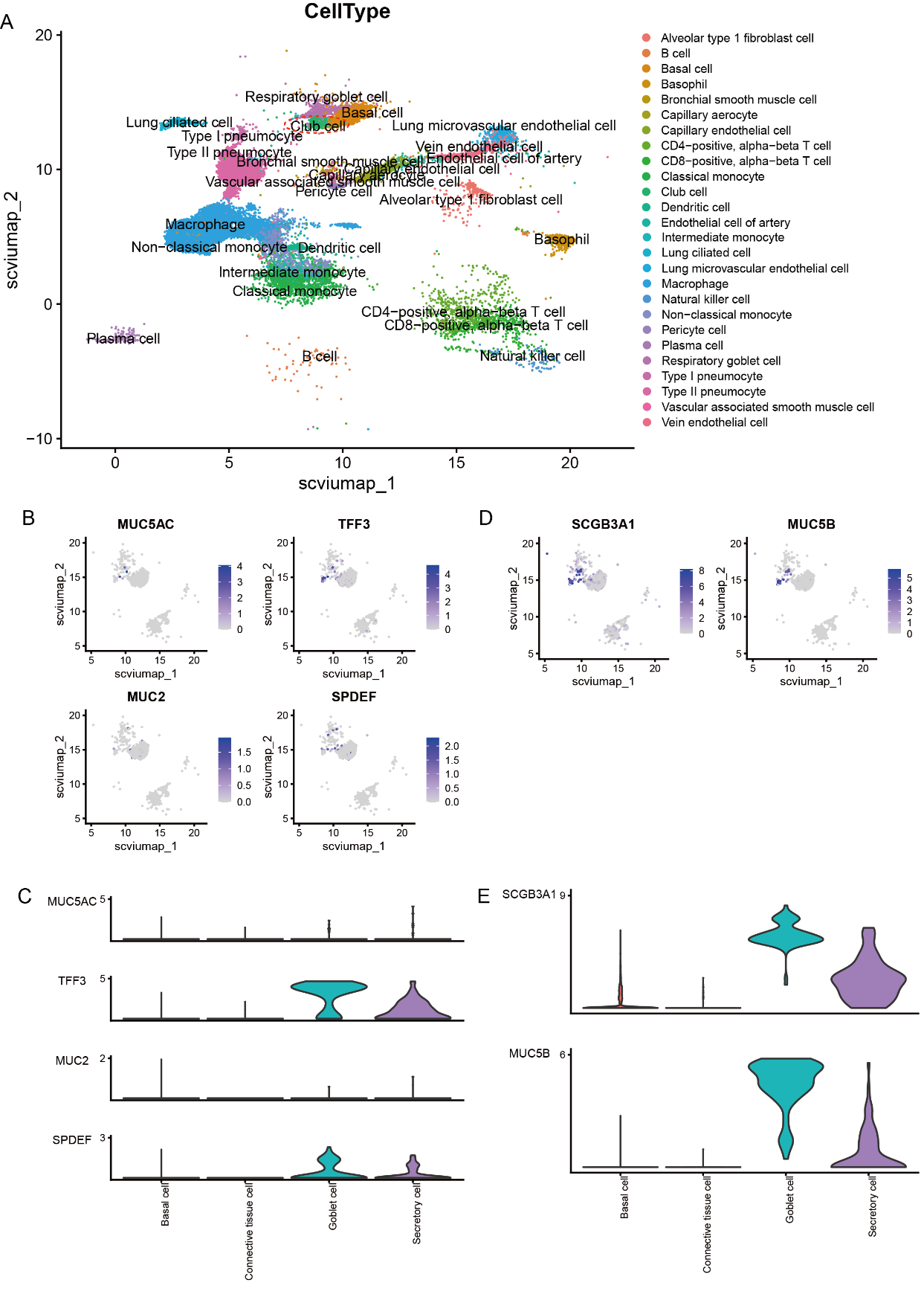


In Tabula Sapiens Muscle, the cluster labeled as "Mesenchymal stem cell" may have been inaccurately annotated previously (A), as it exhibited high expression of the Fibroblast markers, such as DCN, LUM, PDGFRA, and FBLN1 (D and E), while showing low expression of markers for Mesenchymal stem cell, including THY1, NT5E, POU5F1, and SOX2 (C and B).


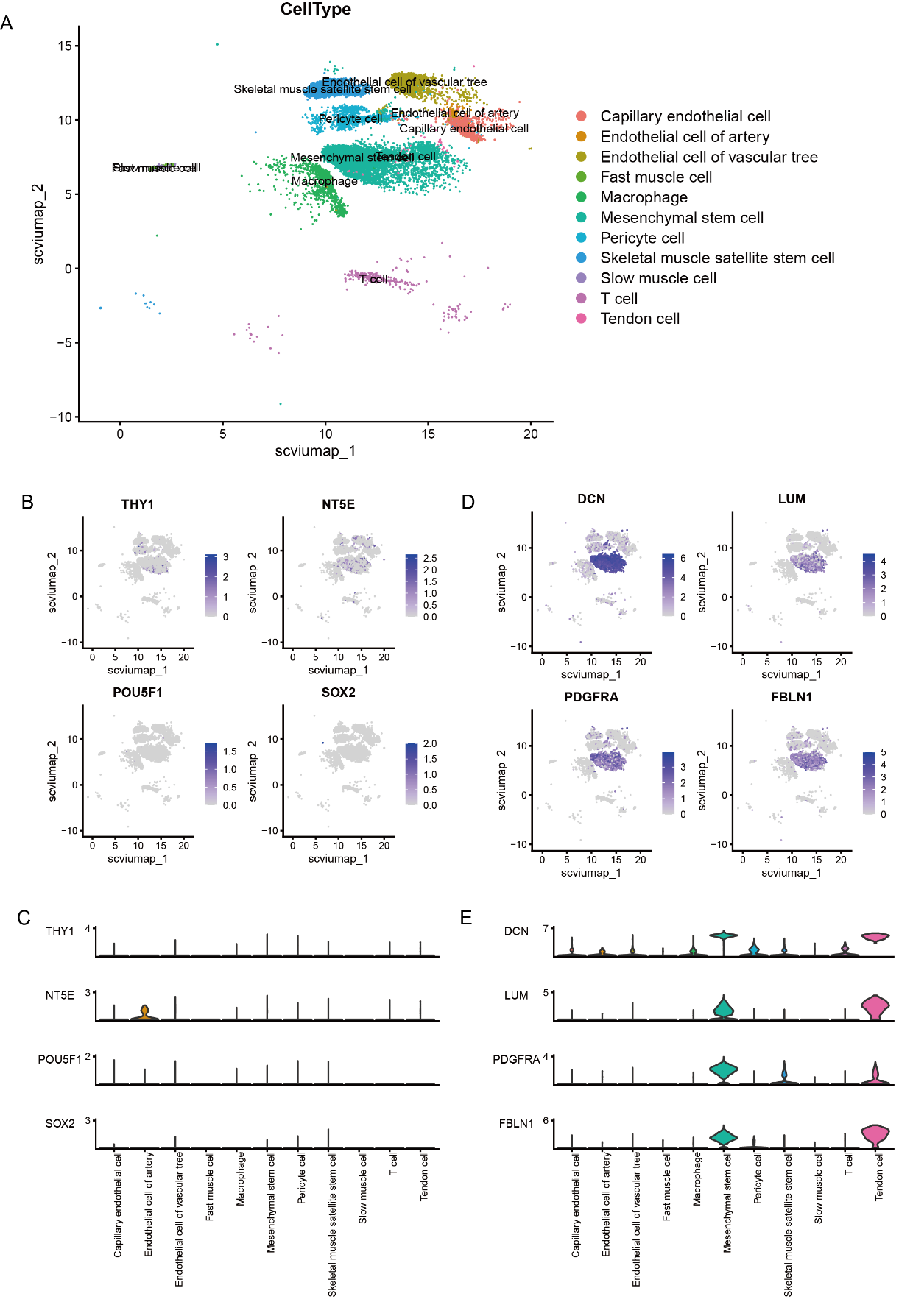


In Tabula Sapiens Muscle, the cluster labeled as "Pericyte cell" was found to be a mixture of Pericyte cell and Smooth muscle cell (A). This cluster was determined to represent a mixture of both Pericyte cell and Smooth muscle cell, exhibiting high expression of the Pericyte cell markers like ACTA2 and RGS5 (C and B), as well as elevated expression of Smooth muscle cell marker, TAGLN and MYH11 (D and E).


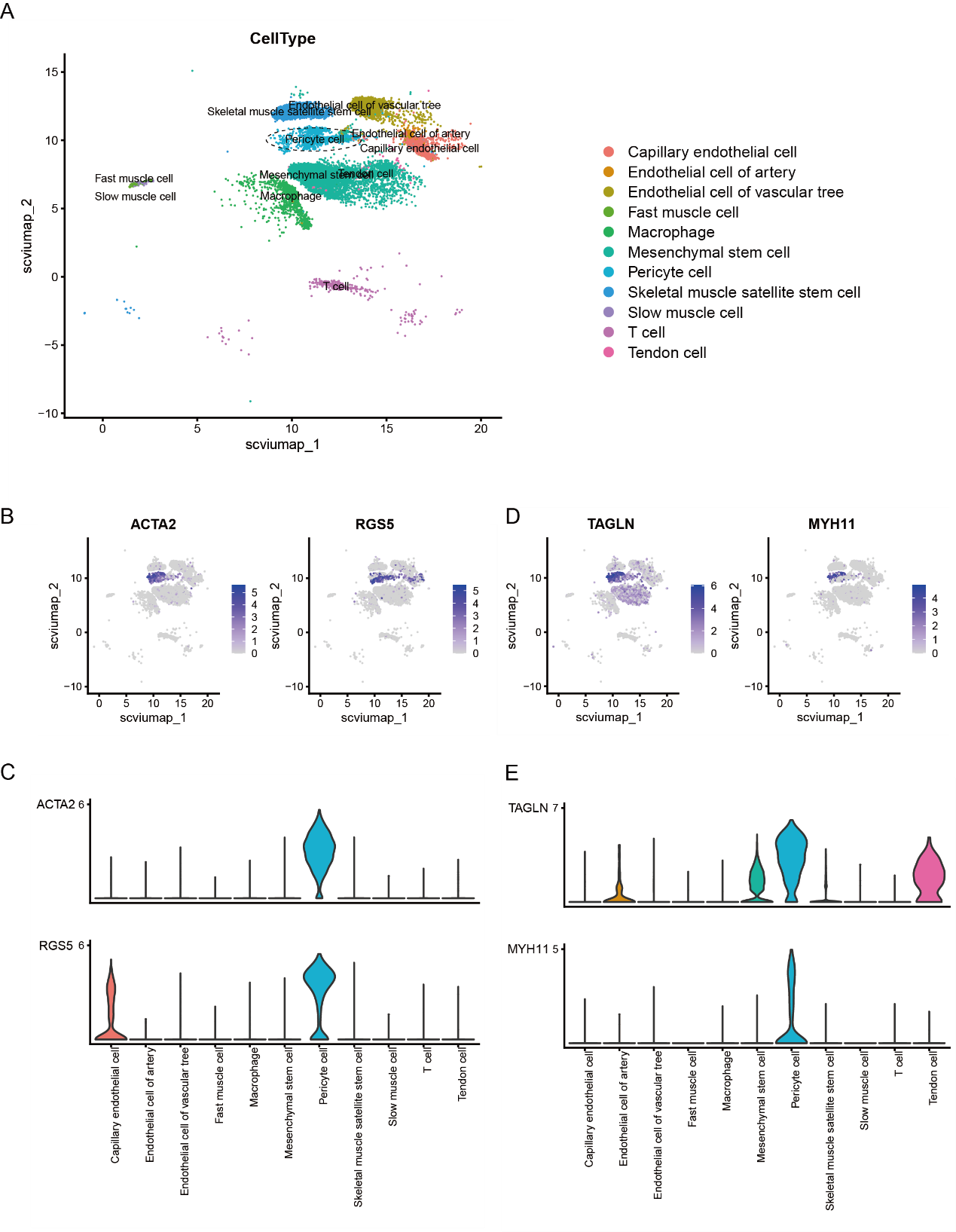


In Tabula Sapiens Spleen, the cluster labeled as "Plasma cell" was found to be a mixture of Plasma cell and Natural killer cell (A). This cluster was determined to represent a mixture of both Plasma cell and Natural killer cell, exhibiting high expression of the Plasma cell markers like JCHAIN and MZB1 (C and B), as well as elevated expression of Natural killer cell marker, GNLY, NKG7, KLRF1 and KLRD1 (D and E).


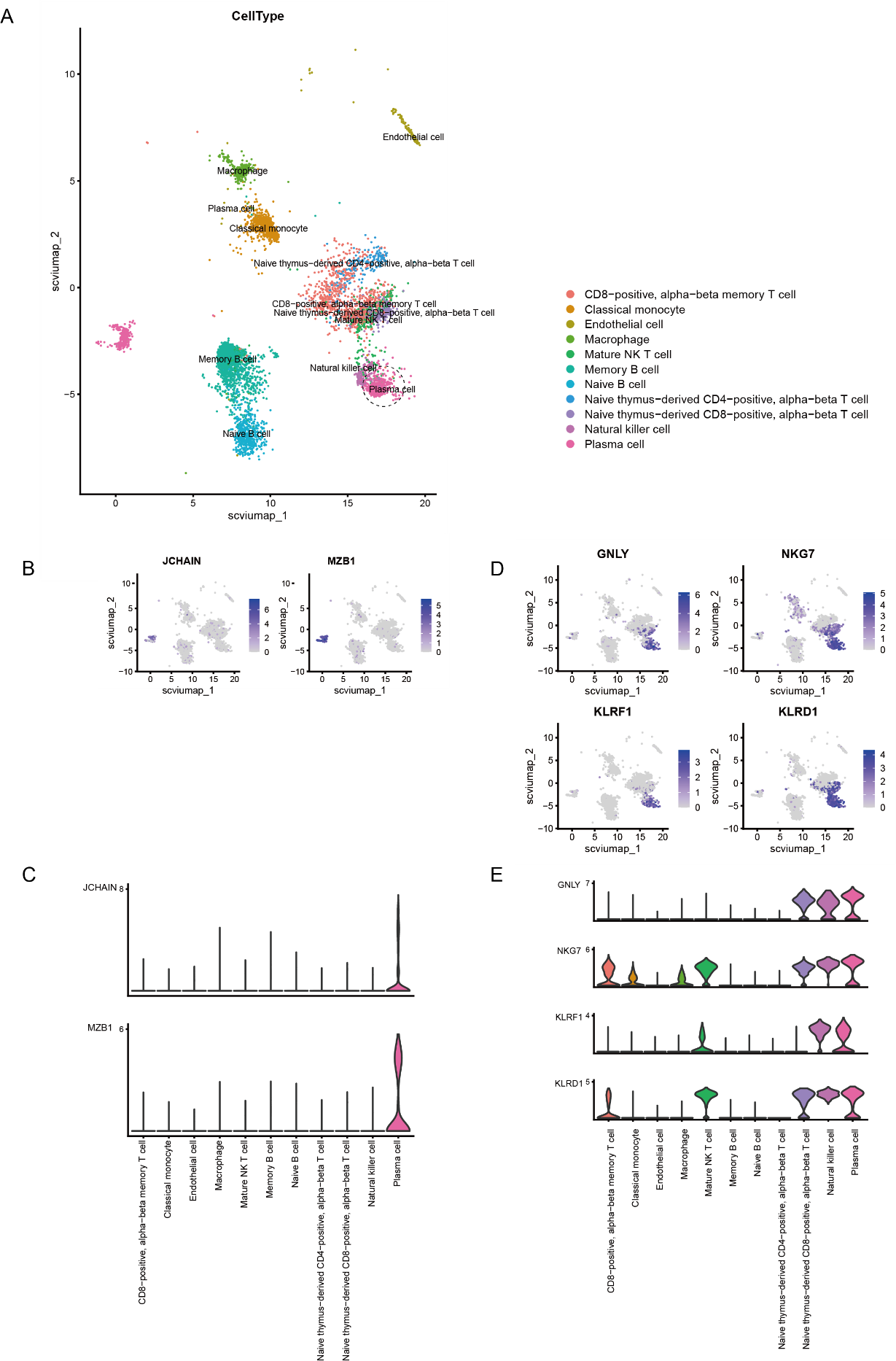


In Tabula Sapiens Spleen, the cluster labeled as "Naive thymus-derived CD8-positive, alpha-beta T cell" may have been inaccurately annotated previously (A), as it exhibited high expression of the CD8-positive, alpha-beta cytotoxic T cell markers, such as FGFBP2, GNLY, KLRD1 and PRF1 (D and E), while showing low expression of markers for Naive thymus-derived CD8-positive, alpha-beta T cell, including LEF1, SELL and TCF7 (C and B).


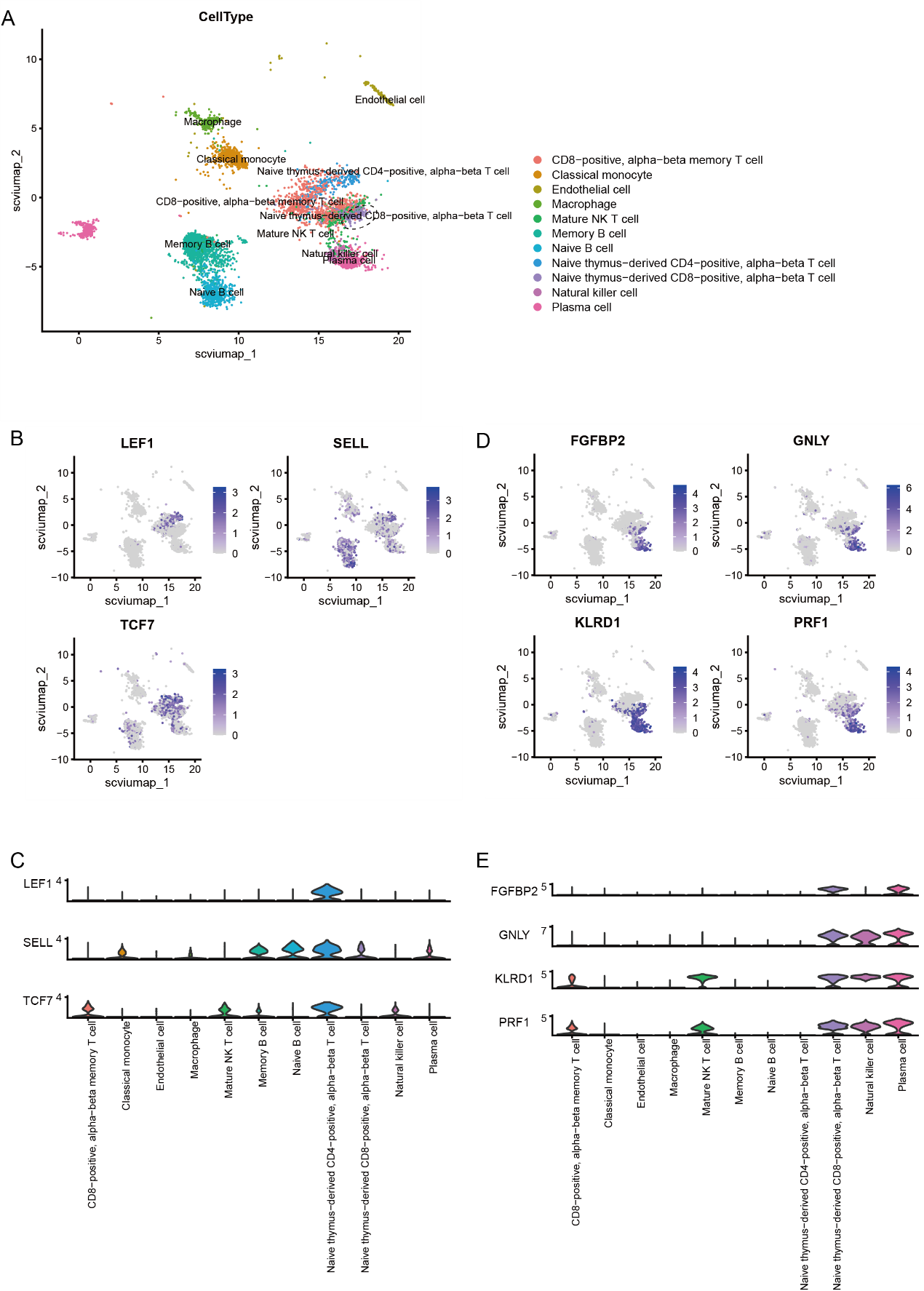


In Human Liver, the cluster 2 was a subgroup of "Kupffer cell". This cluster was found to be a mixture of Kupffer cell and Dendritic cell (A), exhibiting high expression of the Kupffer cell markers like CD163, C1QA and C1QC (C and B), as well as elevated expression of Dendritic cell marker, ITGAX, CLEC10A and CLEC9A (D and E).


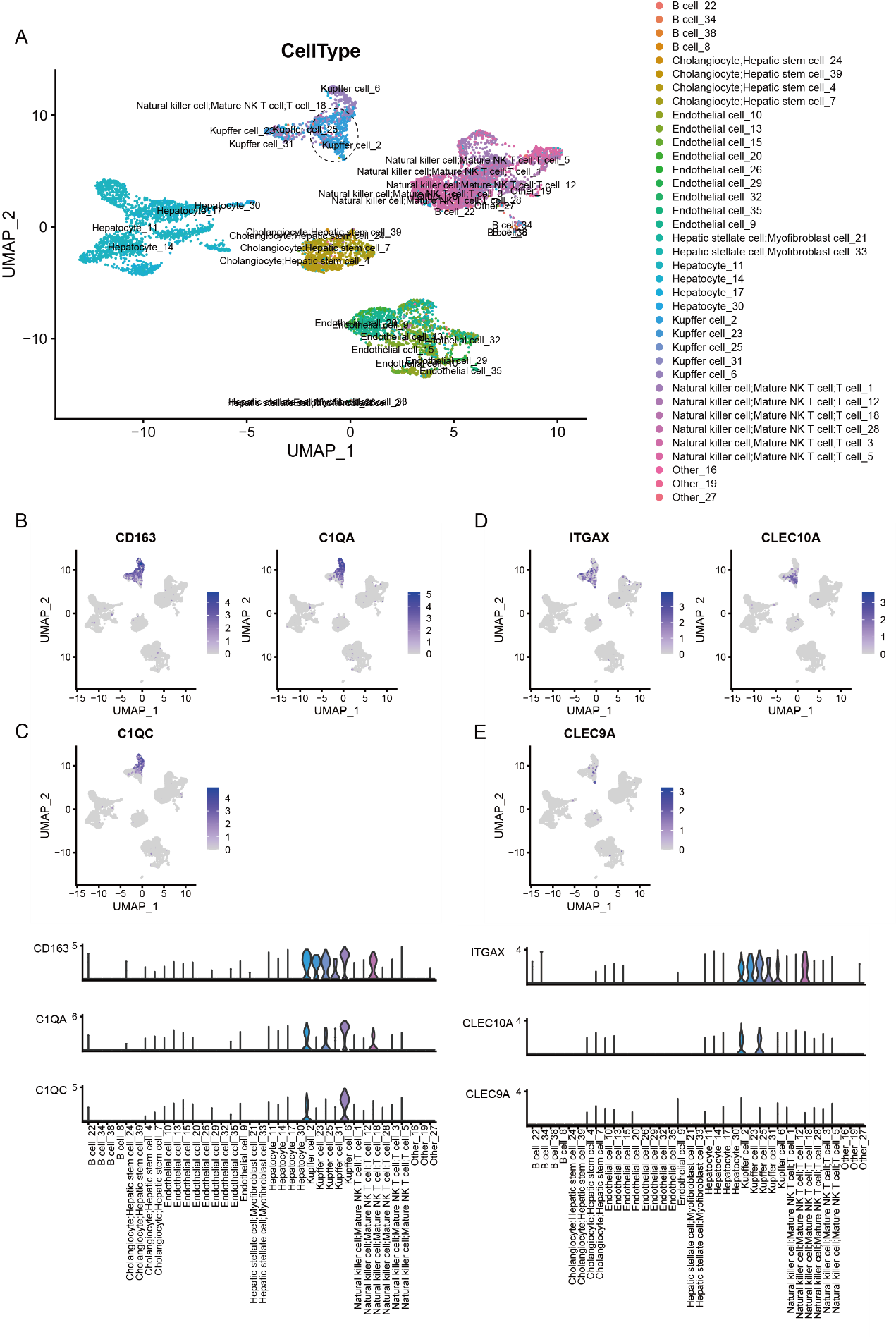


In Human Liver, the cluster 23 was a subgroup of "Kupffer cell".  This cluster was determined to represent a mixture of both Kupffer cell and Monocyte (A), exhibiting high expression of the Kupffer cell markers like CD163, C1QA and C1QC (C and B), as well as elevated expression of Monocyte marker, VCAN and FCN1 (D and E).


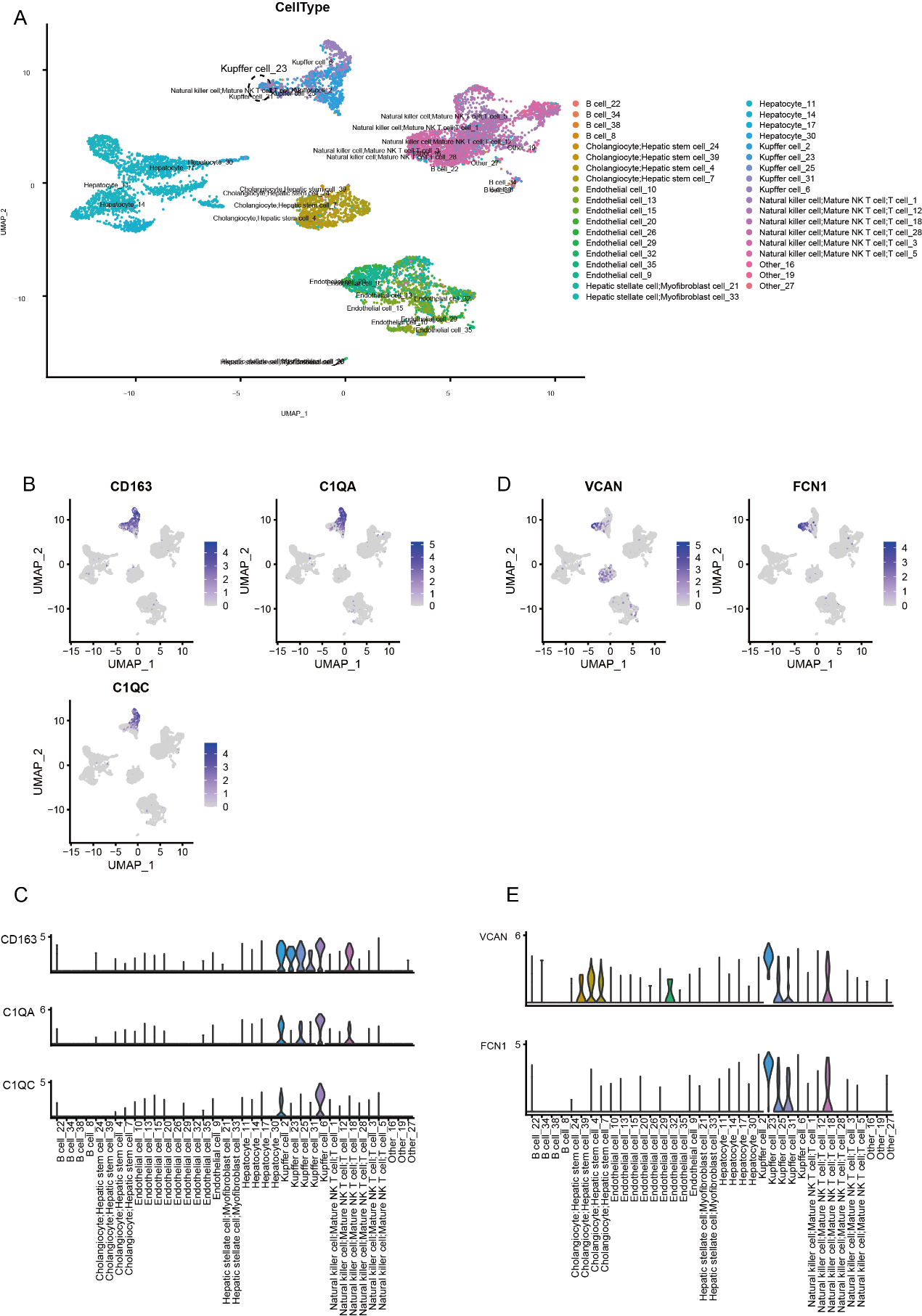


In Human Liver, the cluster 25 was a subgroup of "Kupffer cell".  This cluster was determined to represent a mixture of both Kupffer cell and Dendritic cell(A), exhibiting high expression of the Kupffer cell markers like CD163, C1QA and C1QC (C and B), as well as elevated expression of Dendritic cell marker, ITGAX and CLEC10A (D and E).


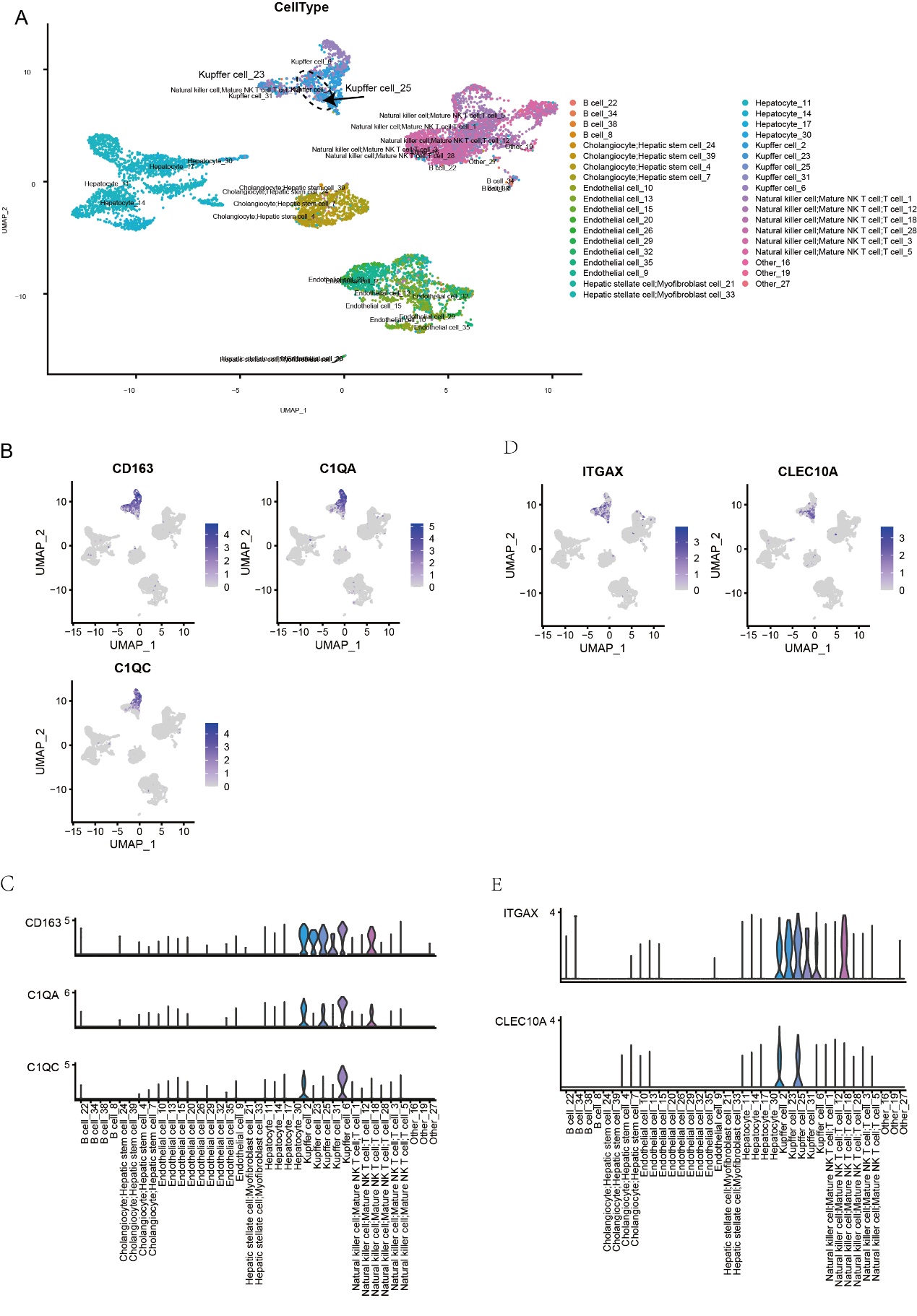


In Human Liver, the cluster 18 labeled as "Natural killer cell; Mature NK T cell; T cell" may have been inaccurately annotated previously (A), as it exhibited high expression of the Monocyte markers, such as FCN1, VCAN and LYZ (D and E), while showing low expression of markers for Natural killer cell; Mature NK T cell; T cell, including NKG7, CD3E, CD3D, and CD3E (C and B).


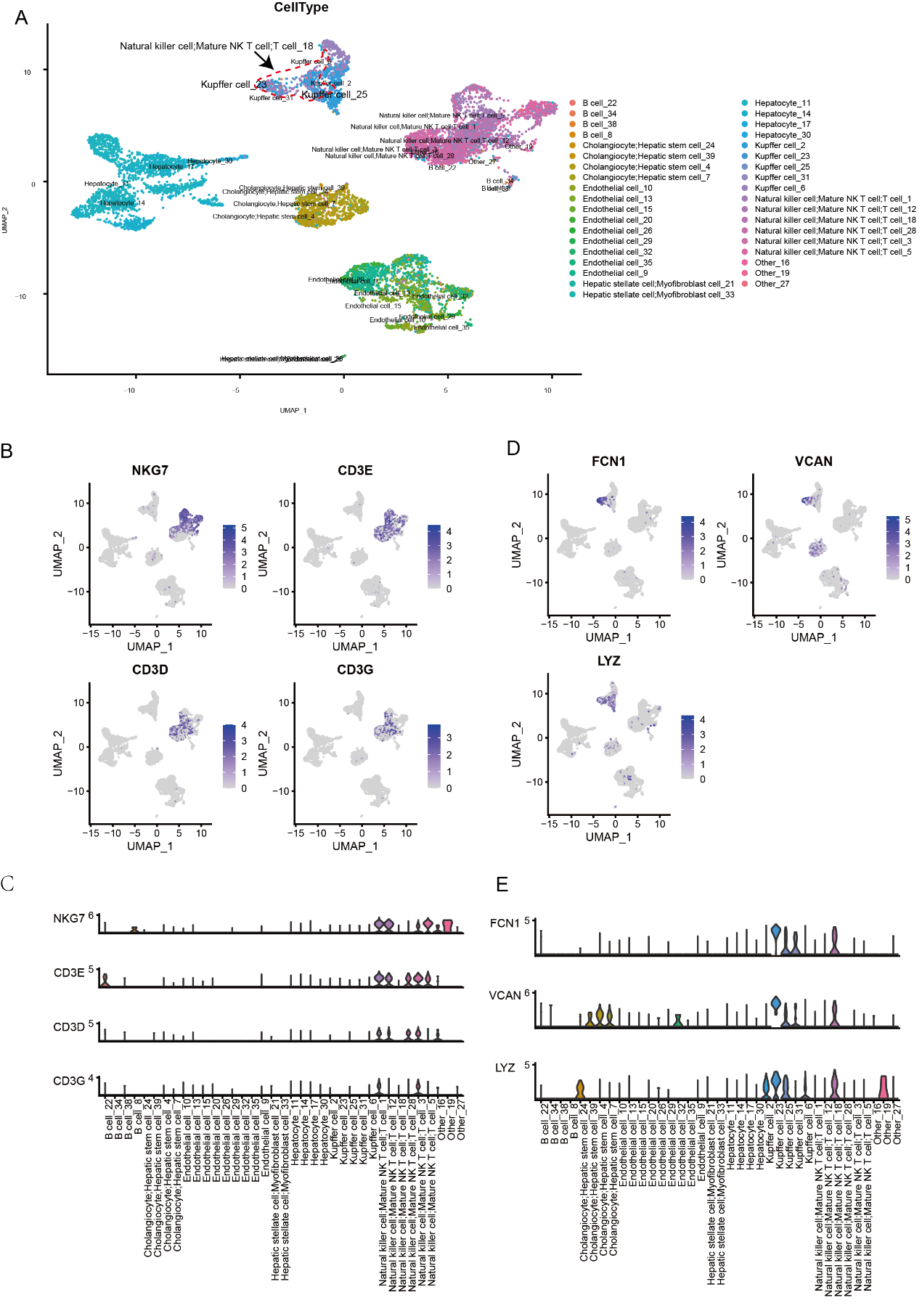


In Mouse Lung, the cluster labeled as " Lung goblet cell" may have been inaccurately annotated previously (A), as it exhibited high expression of the Club cell markers, such as Scgb1a1, Cyp2f2, Scgb3a1 and Muc5b (D and E), while showing low expression of markers for Lung goblet cell, including Age2 and Spdef (C and B).


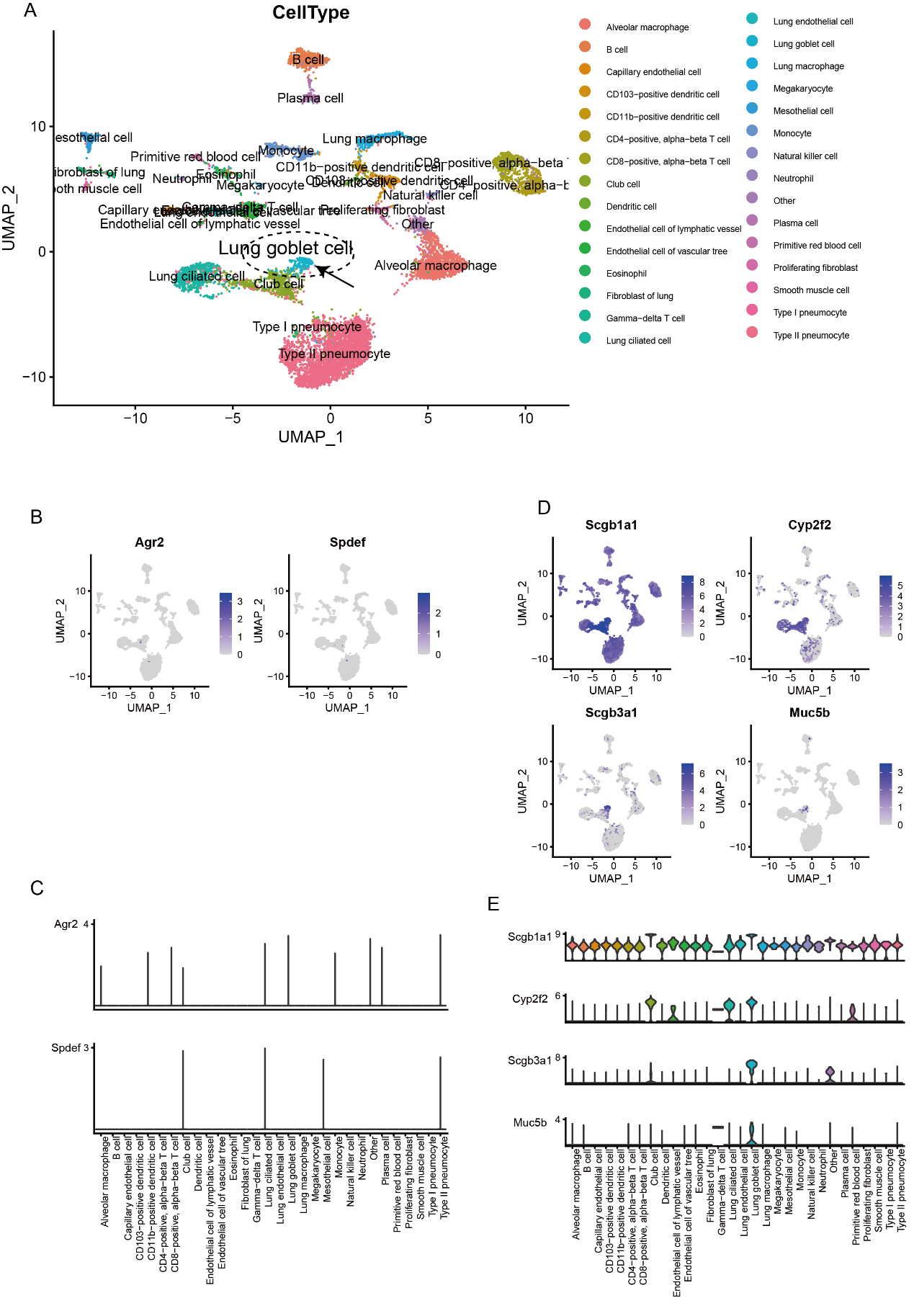


In Mouse Lung, the cluster labeled as "Proliferating fibroblast" may have been inaccurately annotated previously (A), as it exhibited high expression of the Bronchioalveolar stem cells markers, such as Stmn1, Ube2c, Tubb5 and Tuba1b (D and E), while showing low expression of markers for Proliferating fibroblast, including Col1a1, Dcn, Pdgfra, and Lum(C and B).


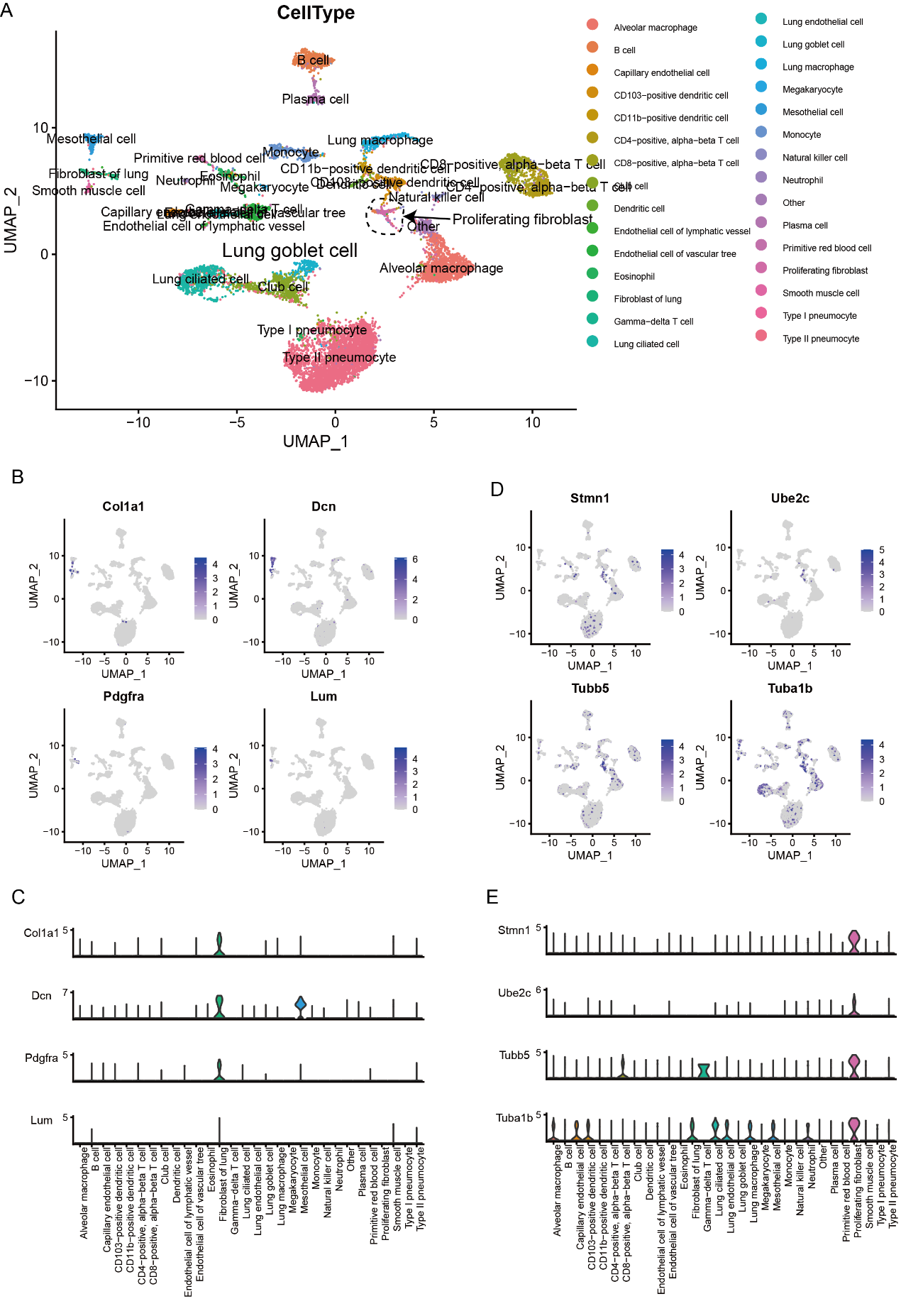

Supplement: Supplementary file 2 — Additional file 2: Fig. S2. Manual inspection and correction of originally incorrectly annotated clusters across five datasets. [file 13073_2023_1249_MOESM2_ESM.docx]
